# Cardiac Cachexia: Unaddressed Aspect in Cancer Patients

**DOI:** 10.3390/cells11060990

**Published:** 2022-03-14

**Authors:** Sarama Saha, Praveen Kumar Singh, Partha Roy, Sham S. Kakar

**Affiliations:** 1 Department of Biochemistry, All India Institute of Medical Sciences, Rishikesh 249203, India; saramasaha@yahoo.co.in (S.S.); praving92@gmail.com (P.K.S.); 2 Department of Biotechnology, Indian Institute of Technology Roorkee, Roorkee 247667, India; paroyfbs@iitr.ac.in; 3 Department of Physiology and Brown Cancer Center, University of Louisville, Louisville, KY 40292, USA

**Keywords:** cardiac cachexia, cancer, angiotensin II, chemotherapy, autophagy, proinflammatory cytokines, withaferin A

## Abstract

Tumor-derived cachectic factors such as proinflammatory cytokines and neuromodulators not only affect skeletal muscle but also affect other organs, including the heart, in the form of cardiac muscle atrophy, fibrosis, and eventual cardiac dysfunction, resulting in poor quality of life and reduced survival. This article reviews the holistic approaches of existing diagnostic, pathophysiological, and multimodal therapeutic interventions targeting the molecular mechanisms that are responsible for cancer-induced cardiac cachexia. The major drivers of cardiac muscle wasting in cancer patients are autophagy activation by the cytokine-NFkB, TGF β-SMAD^3^, and angiotensin II-SOCE-STIM-Ca^2+^ pathways. A lack of diagnostic markers and standard treatment protocols hinder the early diagnosis of cardiac dysfunction and the initiation of preventive measures. However, some novel therapeutic strategies, including the use of Withaferin A, have shown promising results in experimental models, but Withaferin A’s effectiveness in human remains to be verified. The combined efforts of cardiologists and oncologists would help to identify cost effective and feasible solutions to restore cardiac function and to increase the survival potential of cancer patients.

## 1. Introduction: Definition of Cancer Cachexia

The burden of cancer is growing rapidly worldwide. According to GLOBOCON 2020, there will be an approximately 47% increase in cancer patients from 2020 to 2040. Cancer is considered to be the leading cause of death worldwide [1]. Cancer induces cachexia, which is associated with muscle weakening and loss. It is a multifactorial syndrome, and almost 20–30% of cancer-related deaths can be attributed to cachexia rather than to the tumor itself. This is a debilitating and demoralizing condition that is experienced by a majority of the patients, especially during the late stages of cancer (almost 80%). The prevalence of cachexia varies depending on the type of cancer. Patients with tumor masses originating from the pancreas, colon, and stomach experience greater weight loss compared to patients who have been diagnosed with other types of cancer [2]. This muscle wasting not only makes the person weak, leading to poor quality of life, but also contributes to the refractoriness of chemotherapy, and to reductions in overall survival [3]. 

In addition to muscle wasting, cancer cachexia is a devastating syndrome that is characterised by decreased food intake and altered metabolism, giving rise to a negative balance between protein synthesis and energy consumption. Cancer cachexia is described by Fearon K et al. [4] as a “multifactorial syndrome defined by an ongoing loss of skeletal muscle mass (with or without loss of fat mass) that cannot be fully reversed by conventional nutritional support and leads to progressive functional impairment” [4]. There has been a consensus on the accepted key features for the diagnosis of cancer cachexia, which are “weight loss greater than 5%, or weight loss greater than 2% in individuals already showing depletion according to current body weight and height (body-mass index [BMI] <20 kg/m^2^) or skeletal muscle mass (sarcopenia)”. Moreover, this consensus also agrees that a cancer patient goes through a spectrum of cachexia “(pre-cachexia to cachexia to refractory cachexia)” [4]. Muscle wasting is of great importance in poor quality of life, decreased survival, and resistance to therapeutic response [3]. Most of the research on cancer cachexia is primarily focused on identifying mechanisms and targeting therapies towards the skeletal muscle. Recent developments have revealed that cancer cachexia is not only restricted to skeletal muscle dysfunction, but it also affects various other organs, and it is therefore presented as systemic phenomena [5].

## 2. Multiple Organ Involvement in Cancer Cachexia

In addition to muscle wasting, the depletion of adipose tissue and its extensive remodelling, including enhanced resting energy expenditure and free fatty acid degradation, have been documented in patients with gastrointestinal cancer [6]. Moreover, it has also been documented that lipolysis occurs prior to muscle wasting in some cases. Hence, adipose tissue mass could be considered as a better predictor for overall survival in those progressive cancer cachexia patients [7]. Hence, lipolysis is accepted as a novel therapeutic target to counteract progressive wasting in cancer cachexia.

Other than the skeletal muscle and adipose tissue, the dysregulation of the hepatic metabolism might play a critical role in determining the cachectic phenotype in cancer patients. The disruption of the ATP synthesising machinery following the hyperactivation of cardiolipin expression documented in the mitochondria of cachexia-inducing tumor-bearing mice might explain the reason for the protein energy imbalance observed in cachexia [8]. It was reported that fenofibrate, the peroxisome proliferator-activated receptor alpha (PPARα) agonist, [9] could revert the ketogenic potentiality back to what it was originally in a tumor-bearing experimental animal model. In addition, the documentation of abnormal liver function tests along with the induction of the hepatic acute phase response (APR) in pancreatic cancer patients [10] as well as observations of enhanced hepatic gluconeogenesis documented by the infusion of ^14^C-labelled glucose in colorectal cancer patients [11] further emphasizes the significant contribution of liver metabolism in the etiopathogenesis of cancer cachexia.

**Figure 1:**
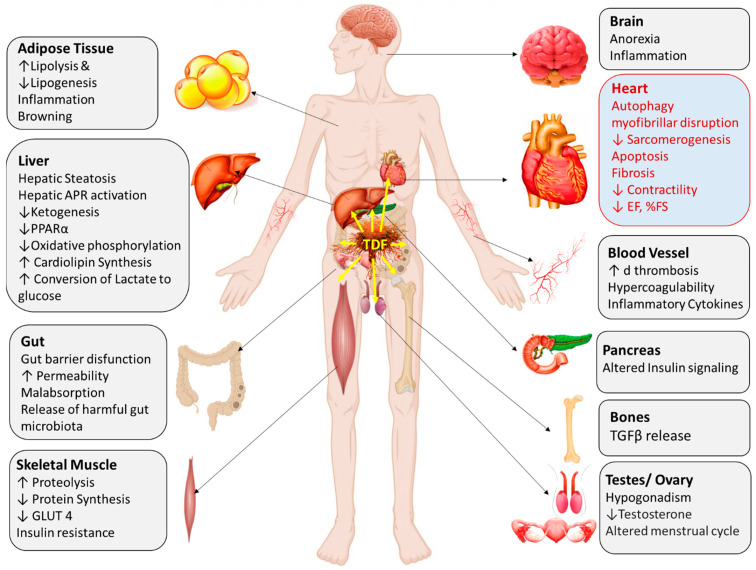
Cancer-induced cachexia involves multiple organs, including the heart and is a systemic phenomenon. GLUT: glucose transporter, APR: acute phase response, PPAR: peroxisome proliferator-activated receptors, EF: ejection fraction, FS: fractional shortening, TDF: tumor-derived factor. “↑” denotes increase. “↓” denotes decrease. Redrawn with modification from ref [5].

In addition, the alterations in the functions of various other organs, such as the heart, brain, pancreas, intestine, testes, and ovaries, also contribute to the pathophysiology of cancer-induced cachectic features [5] (Figure 1). It has been documented that the brain suppresses appetite through the disruption of the orexigenic pathway and by modulating neuropeptide Y release [12]. The documentation of a decreased abundance of lactobacillus species in an experimental mice model and the demonstration of skeletal muscle wasting via gut microbiota-derived mediators and the modulation of Ghrelin release provide evidence of intestinal involvement in the etiology of cachectic features in cancer patients [13]. Preventing the progression of cachectic symptoms in inhibin-deficient mice via the suppression of the circulating functional activin level via the administration of chimeric activin receptor II joined to the Fc region of a murine immunoglobulin indicated that the gonads contribute to the cachectic phenotype through the FSH regulators activin and inhibin [14]. 

The inflammatory mediators originating from the host and the tumor microenvironment are transported to the site of the action by the circulatory system, which acts as a link between the different tissues that are involved in cachexia. Moreover, the hypercoagulability and thrombocytosis that are documented in tumor-bearing experimental animal and clinical cases might provide a plausible explanation for the increased mortality in cancer cachexia [15]. Hence, cancer cachexia is the consequence of an imbalance in the catabolic and anabolic pathways resulting from the complex interplay between disturbed immunological, metabolic, neurohormonal and genetic factors. In addition to affecting quality of life, this progressive wasting disorder could alter the responsiveness to the chemotherapy and increase the toxicity of the administered pharmacotherapies, enhancing the risk of complications after surgical intervention and resulting in a poor prognosis while also increasing mortality in cancer patients [2]. 

Despite the recent advances that have been made in cancer management, little attention has been paid to the cancer-induced cardiac dysfunction, which might further aggravate the existing process that is involved in losing body weight. Hence, this review would address the etiopathogenesis as well as the existing diagnostic and therapeutic approaches of cancer-induced cardiac cachexia to provide a comprehensive understanding of the heterogeneity of this devastating condition. We have also discussed how chemotherapy aggravates cardiac dysfunction in cancer patients and have described both preclinical and clinical studies.

## 3. Definition and Pathophysiology of Cancer-Induced Cardiac Cachexia

Cardiac cachexia is defined as a dysfunction of the heart in a patient experiencing cardiac muscle wasting due to a catabolic shift of the dysbalanced metabolic homeostasis. The development of cachexia is facilitated by the coexistence of metabolic abnormalities and prolonged heart failure and has become a progressively growing public health issue, affecting nearly 26 million people around the world [16]. It has been documented that heart failure patients presenting with cachexia have a poorer prognosis compared to non-cachectic individuals and that the condition has negative effects on quality of life [17]. Cancer patients might develop cardiac abnormalities such as a decrease in left ventricular mass, the impairment of cardiac output, alterations in diastolic function, reductions in the size of cardiac myocytes, and the induction of cardiac fibrosis. [18]. Therefore, it is of the utmost important to diagnose the cause of cachexia, including the cardiac component, as early as possible to provide adequate time to the clinician to treat the condition and to reduce the mortality resulting from progressive complications [19]. 

### 3.1. Inflammation and Immune Modulation

The pathophysiology of cardiac cachexia involves the immune activation of the cytokine cascade, which includes the augmented release of inflammatory cytokines such as tumor necrosis factor alpha (TNF-α), interleukin-1β (IL-1β), and interleukin-6 (IL-6) as well as a decrease in anti-inflammatory cytokines such as IL-10 and cytokines that have pleiotropic effects on cellular differentiation, such as transforming growth factor beta 1 (TGF-β1) [20,21]. TNF- α is hypothesised to be released by the failing heart itself, or it may be released from extra myocardium because of tissue hypoxia resulting from peripheral vasoconstriction. The TNFα serum level is inversely proportional to the peripheral blood flow [22]. However, TNFα induces proteolysis through the up-regulation of the ubiquitin–proteasome cascade via the modulation of the NFκB pathway [23]. TNF-α exhibits its harmful and toxic effects via the TNF receptor 1 and its beneficial effects via TNF receptor 2 [24]. TNF- α, along with another cytokine, IL-1β, impede beta the adrenergic response of the cardiac myocytes [25]. TNF-α also induces the release of IL-6, which is implicated in the pathophysiology of cardiac cachexia via the induction of an acute phase response and is supported by high circulating levels of IL-6 in heart failure patients [26]. Proinflammatory cytokines also promote weight loss by inhibiting the synthesis of anabolic hormones such as insulin-like growth factor (IGF-1). TGF-β and its family members, which comprise activins, myostatin, and the growth and differentiation factor 11 (GDF11), induce cardiac atrophy and fibrosis through the phosphorylation of the SMAD proteins (canonical pathway) or via the involvement of the ERK and p38/MAP kinase pathway (noncanonical). However, they initiate signalling cascade via the activation of activin type II receptors A and B (ActRIIA and ActRIIB), which are located on cardiac myocytes. Binding with ActRII induces the phosphorylation of the downstream targets SMAD 2 and 3, which form a heterodimer complex. It then incorporates SMAD 4 and forms a trimer that translocates into the nucleus and influences the transcription of the target gene. Activin further inhibits protein synthesis via the inhibition of the Akt/mTOR pathway. It promotes protein degradation via the increased translocation of FOXO3 in the nucleus followed by the enhanced expression of the ubiquitin ligase [27].

### 3.2. Hormones and Proteolytic Pathways

Metabolism alterations that are in favor of the catabolism observed in cardiac cachexia are triggered by upregulated norepinephrine and epinephrine and result in augmented resting energy dissipation [28]. A disproportionate increase in the glucocorticoid level may be responsible for cardiac cachexia by reducing the protein synthesis and by augmenting proteolysis. Protein synthesis is affected by the down regulation of insulin-like growth factor 1 (IGF1) and myogenin expression. The IGF1-mediated activation of Akt/mTOR inhibits proteolysis. Hence, the down regulation of IGF1 may contribute to a decrease in myogenesis via the deactivation of the mTOR pathway [29].

Cosper and Leinwand [30] documented the progressive hyperactivation of cathepsin D, Cathepsin L, and microtubule-associated protein 1 light chain 3 (LC3 I and LC3 II), which are indicators of lysosomal activation in the cardiac muscle of tumor-bearing mice, indicating the contribution of the autophagic pathway during the proteolysis of the cardio myocytes resulting in cancer-induced cardiac atrophy [30]. The involvement of muscle ring finger 1 (MuRF1) in ubiquitin ligase activity was documented to degrade the sarcomeric protein (troponin I) in an experimental mice model of cardiac atrophy [31]. Surprisingly, Cosper and Leinwnand [30] could not determine the involvement of (Atrogin-1 and MuRF-1) in the protein degradation of the cardiac muscles in a cancer cell-inoculated animal model. 

**Figure 2:**
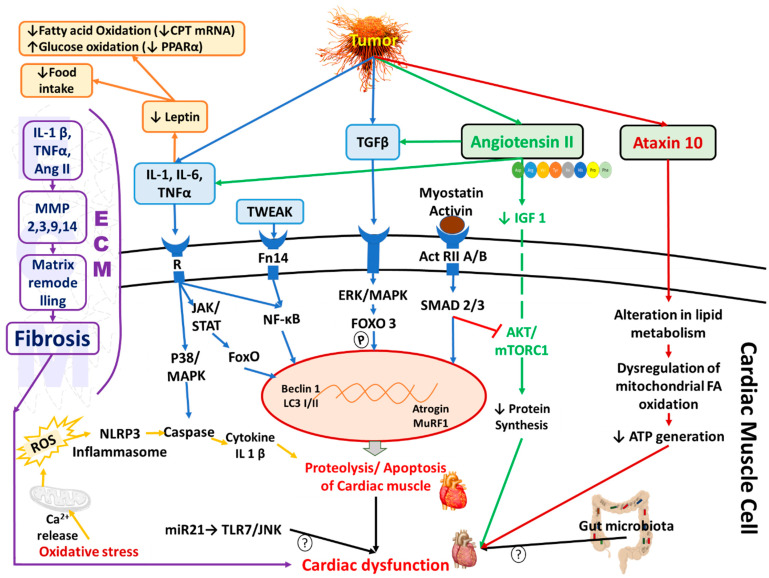
Schematic diagram showing the molecular mechanisms underlying cardiac cachexia in cancer patients. Some pathways, such as the cytokine, myostatin, activin, and leptin-mediated signalling pathways, are involved in both skeletal and cardiac muscle wasting. IL: interleukin, ROS: reactive oxygen species, NFkB: nuclear factor kappa B, IGF1: insulin-like growth factor 1, TNF: tumor necrosis factor, TGF: transforming growth factor, ERK: extracellular signal-regulated kinases, ActRIIA: activin type II receptors A, Foxo: forkhead transcription factors, MuRF 1: muscle ring finger protein-1, LC3: microtubule-associated protein 1A/1B-light chain 3, mTOR: mammalian target of rapamycin, NLRP3: NLR family pyrin domain containing 3, TLR: Toll-like receptor, miRNA: micro RNA, JNK: c-Jun N-terminal kinase, ECM: extra-cellular matrix. “↑” denotes increase. “↓” denotes decrease. Redrawn with modification from reference [27].

Furthermore, a significant decrease in calpastatin and 130 kDa Ca2^+^-ATPase activity in cell membranes isolated from cardiac muscles indicates the involvement of Ca2^+^-dependent protein degradation that results in cardiac atrophy in AH-130 hepatoma cell-induced tumor bearing male Wistar rats [32]. The pathophysiology of the development of cancer-induced cardiac cachexia is presented schematically in Figure 2.

### 3.3. Mitochondrial Dysfunction and Oxidative Stress

An animal model of male C57BL6/J mice inoculated with Lewis Lung Carcinoma cells documented that cancer-induced cardiac atrophy was associated with a significantly lower redox ratio, indicating that the mitochondrial oxidative metabolism was reduced. It was also associated with the induction of a hypoxic state, which was validated by the presence of hypoxia inducible factor 1α (HIF1α) and more dependency on glucose as a substrate. Moreover, a reduction in the total content of the mitophagy protein PTEN-induced kinase 1 (PINK1) and a decrease in the ratio of phosphorylated to total PINK1 indicated that the accumulation of abnormal polarized mitochondria could be one of the etiologies of cardiac cachexia. The inefficient electron flow through complexes I and III resulting from the incorrect incorporation of the OXPHOS subunit by the decreased amount of mtDNA-encoded protein cytochrome b results in the generation of a greater amount of superoxide. Furthermore, significantly lower levels of glutathione peroxidase (GPX)-3 and GPX-7 results in an accumulation of H_2_O_2_ [33]. 

**Figure 3:**
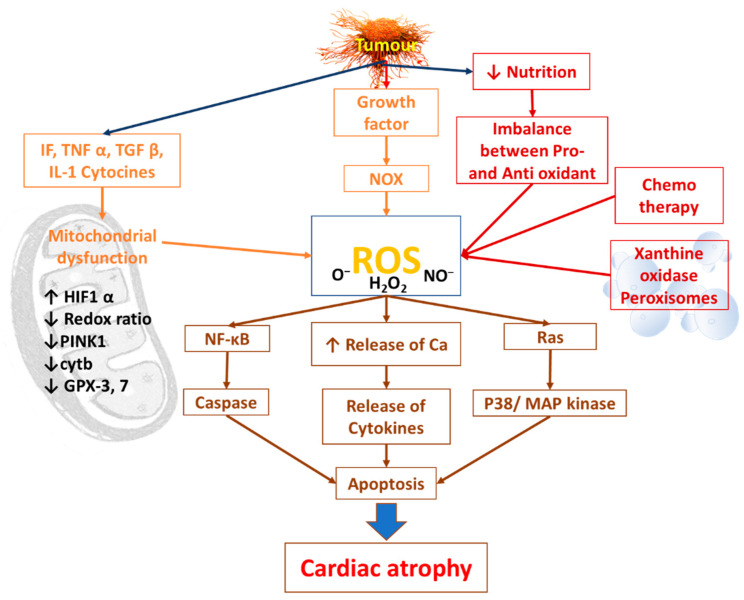
Diagram showing different sources of reactive oxygen species (ROS) and its probable mechanism for the induction of cardiac atrophy in cancer patients. NOX: NADPH oxidase, cyt b: cytochrome b, GPX-3, 7: glutathione peroxidase 3 and glutathione peroxidase 7, HIF1α: hypoxia inducible factor, PINK1: PTEN induced kinase1. “↑” denotes increase. “↓” denotes decrease. Redrawn with modification from ref [37] under Attribution License (CC BY 4.0).

The prevention of cardiac atrophy via the administration of potent antioxidant resveratrol in C26 tumor-bearing mice indicates the crucial role of reactive oxygen species (ROS) in the development of cancer-induced cardiac atrophy [34]. In addition to mitochondrial dysfunction, xanthine oxidase in the peroxisomal matrix and the peroxisomal membranes could be the source of ROS because it is possible to reduce cardiac atrophy via the administration of the xanthine oxidase inhibitor in rats that have been inoculated with AH-130 Yoshida hepatoma cells [35]. Furthermore, a loss of balance between the pro- and anti-oxidants resulting from the inadequate intake of nutritional substances because of anorexia caused by cancer itself or by the chemotherapeutic drugs administered for the treatment of the specific disease could result in ROS accumulation [36,37] (Figure 3). 

## 4. Angiotensin II-Induced Cardiac Dysfunction Mimics Cardiac Cachexia

**Figure 4:**
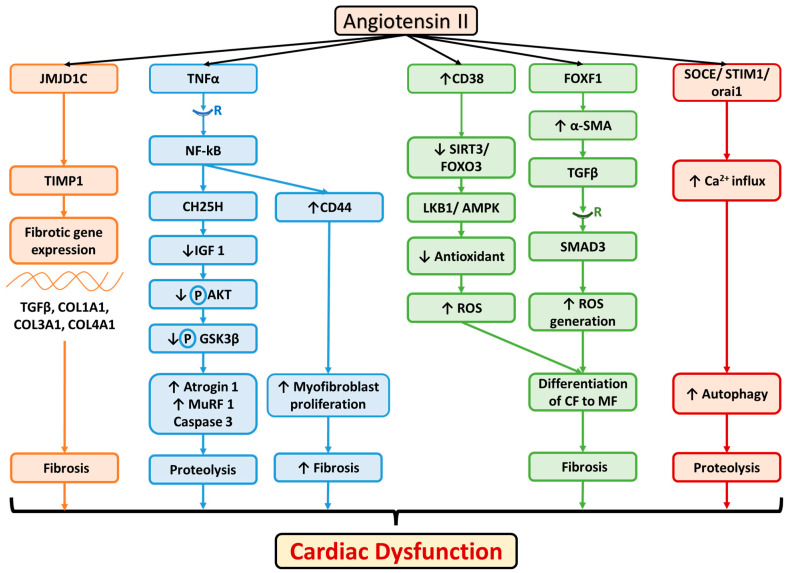
Molecular mechanisms associated with angiotensin II-mediated cardiac dysfunction. CH_25_H: cholesterol 25 hydroxylase, TGFβ: transforming growth factor, CF: cardiac fibroblast, MF: myofibroblast, SMA: smooth muscle actin, SIRT3: sirtuin 3, FOXO: forkhead transcription factors, JMJD1C: Jumonji domain containing 1c; histone demethylase, FOXF1: forkhead box protein F1, LKB1: liver kinase B1, AMPK: AMP activated protein kinase, ROS: reactive oxygen species, SOCE: store-operated calcium entry, STIM: stromal interaction molecule, TIMP: tissue inhibitor of metalloproteinases, R: receptor, COL: collagen, ROS: reactive oxygen species. “↑” denotes increase. “↓” denotes decrease.

Angiotensin II (AngII), the principal mediator of the renin angiotensin aldosterone system, plays a crucial role in cardiovascular homeostasis, and its action primarily takes place through angiotensin II type I receptors (AT1R). AT1 receptor expression is increased in ovarian cancer [38]. A retrospective cohort study conducted on 5207 hypertensive patients in Scotland reported that the prolonged use of angiotensin-I-converting enzyme (ACE) inhibitors showed a protective role against cancer [39]. Later on, in a randomized, double blind, placebo-controlled trial, ACE inhibitors were shown to prevent weight loss in heart failure patients, indicating the putative role of AngII in cancer-induced cardiac cachexia [40]. In an experimental animal model of cardiac cachexia, AngII induced muscle wasting through the downregulation of insulin-like growth factor 1 (IGF1) and the upregulation of ubiquitin-related enzyme atrogin 1 and muscle ring finger 1 (MuRF1) through the TNFα/TNF receptor 1/25 hydroxycholesterol/GSK3β signaling pathway [41,42]. Moreover, AngII plays an important role in cardiac dysfunction through the dysregulation of the oxidative status of the heart through increase in the levels of reactive oxygen species (ROS) [43]. Various preclinical studies documented that AngII mediates myocardial structural protein alterations, such as the overexpression of the β isoform of the myosin heavy chain (MHC) and the skeleton actin protein as well as cardiac fibrosis through the JMJD1C/TIMP 1 [44] and FOXF1/TGFβ 1/SMAD3 [45] signaling pathways as well as the involvement of the CD38/SIRT3 (Sirtuin)–FOXO3, Ca^2+^–calcineurin–NFAT (nuclear factor of activated T cells) [46], and TNFα/NFkB/CD44 signaling pathways [47]. In addition, AngII induces the upregulation of autophagy to mediate its cardiac pathogenesis through the activation of SOCE (store operated Ca^2+^ entry)/Orai1 (a pore forming subunit for calcium permeation)/STIM1(Ca^2+^ sensor) [48] and the inhibition of mammalian sterile 20-like kinase 1 (Mst1), a mediator of the Hippo signaling pathway [49]. In an experimental animal model of heart failure, it was documented that AngII decreased the levels of circulating IGF1 and elevated IGF1 and the expression of its specific receptor in the cardiac muscles [50]. The relative levels of circulating and intra cardiac IGF1 would determine whether AngII-mediated cardiac dysfunction would be primarily associated with cardiomyocyte hypertrophy or cardiac atrophy, which is commonly observed in cardiac cachexia. The biomolecules that are involved in AngII-mediated cardiac dysfunction are summarized in Figure 4.

In nutshell, the cardiac dysfunctions that occur in cancer patients are primarily due to a catabolic shift in metabolic homeostasis, resulting in the aggravation of the proteolysis of the cardiac myocytes. This process is facilitated by inflammation and immune modulation via various cytokines such as IL-6, IL-1, TNFα, and TGFβ as well as mitochondrial dysfunction, resulting in inadequate energy production and the accumulation of an excess amount of reactive oxygen species. Moreover, increased circulating levels of AngII in cancer cachectic patients might play an important role in cardiac dysfunction by decreasing the circulating IGF1 and MMP activation. 

The concomitant and interlinked changes that occur in the skeletal and heart muscles in cancer patients are due to the similar biological drivers (such as inflammatory state, alterations in glucose and insulin metabolism, anemia triggered by iron deficiency) that are shared by these organs, giving rise to the complex cross talk between cachexia and cardiac dysfunction. Skeletal muscle wasting and cardiac abnormalities are bidirectional in cancer patients. The amelioration of cardiac function by improving cancer cachexia via physical exercise and nutritional supplementation could emphasize the importance of the inter-tissue connection in the pathogenesis of cancer-induced cardiac achexia [51]. 

## 5. Diagnosis of Cardiac Cachexia

Despite the increasing prevalence and being a cause of poor quality of life, the diagnosis of cardiac cachexia continues to be a great challenge for clinicians and the research community. Since cachexia is a complex multifactorial syndrome, it would not be possible to identify all of the disorders at the same time with only one investigation. Different parameters that are usually used to assess cardiac function include the ejection fraction (EF) as well as fractional shortening (FS) for left ventricular systolic function and E/A (E: peak early diastolic mitral inflow velocity) as well as E/e’(e’: mitral annulus early diastolic velocity) for diastolic function [18] as well as heart rate variability within 24 h by Holter monitoring and the maximum oxygen uptake VO_2_ peak for exercise capacity, which is directly related to cardiac output by Fick’s law [Cardiac output = O_2_ uptake (VO_2_)/Arteriovenous O_2_ difference (A-VO_2_) [52]. Muscle turnover can be assessed by measuring surrogate marker and urinary creatinine. Most of the investigations carried out to measure the structural and functional status of the cardiovascular system include electrocardiography and echocardiography. A more sophisticated approach is cardiac magnetic resonance imaging [27]. On the other hand, muscle mass can be estimated by imaging techniques such as chest x-ray, dual energy x-ray absorptiometry (DEXA) scans, CT, positron emission tomography (PET), and magnetic resonance imaging (MRI) [53]. Although DEXA scans are a user friendly procedure and are considered to be the gold standard method for the estimation of body composition, it is not reliable in case of cardiac cachexia because of interference caused by retained body water [54]. Other methods such as CT and MRI are very expensive and require skilled individuals to operate the instrumentation as well as to interpret the results. Muscle mass measured in a rat model using the D_3_ creatine dilution method helped to assess skeletal muscle mass following the oral administration of a small amount of deuterated creatinine and in the collection of the first morning sample 48 h after administration. The advantage of this non-invasive method is that the presence of edema, which is frequently accompanied by cachexia in individuals, does not influence the results [55]. One research project plans to measure the skeletal muscle mass in colorectal cancer patients using the D_3_ creatine dilution method, and this may provide information for the clinical usefulness of this technique (NIH RePORTER Project 5R01CA240394-02). 

Various biomarkers that are direct or indirect determinants of the complex phenotypes of cardiac cachexia will help in the early diagnosis of the underlying disease as well as assist in monitoring the response to the administered therapies. Hormone markers comprise of anabolic (insulin growth hormone and IGF1) catabolic factors (epinephrine, norepinephrine, and cortisol), and those related to the cardiovascular system are angiotensin II and atrial natriuretic peptide (ANP) as well as brain natriuretic peptide (BNP). All of these biomarkers are upregulated, except for IGF1 [56,57]. However, Delafontaine and Brink [50] documented the overexpression of IGF1 in the cardiac muscle of an experimental model, and Penna et al. [58] showed that muscle wasting in C26 tumor-bearing mice was not due to the downregulation of IGF1 activity. The discrepancy in finding, the IGF1 level might limit its use as a biomarker for cardiac cachexia. The elevation of the circulating cardiac troponin I [59], the most commonly used biomarker to predict cardiac damage, and alteration of the circulating lipid profile observed in patients with cancer cachexia and chemotherapy-induced cardiac toxicity may be considered to be biomarkers that can be used to gain insight into the cardiovascular status of cancer patients with a cachectic phenotype. Significant increases in activin A levels in lung and colorectal cancer patients with cachexia [60] as well as in heart failure patients [61] reinforce the potential role of activin as a biomarker for cancer-induced cardiac dysfunction. Inflammatory markers include TNF-α, IL-1β, and IL-6. Oxidative stress markers include serum uric acid [62,63,64]. Elevated C reactive protein and decreased haemoglobin and serum albumin levels are also considered to be biological markers for cardiac cachexia [19]. Moreover, the significant correlation between body weight loss and increased serum ataxin-10 levels suggests that ataxin-10 could be considered to be a potential biomarker for diagnostic and or prognostic purposes in colon cancer [65]. 

In summary, considering the multifactorial pathogenesis of cachexia, only one highly sensitive, specific biomarker would not serve the purposes of diagnosis, prognosis, and therapeutic response. A panel of biomarkers would be the ideal solution to this problem. 

## 6. Cancer Induced Cardiac Cachexia: Experimental and Clinical Evidence

It is known that the in some cases, cancer induces cardiac dysfunction that eventually leads to chronic heart failure (CHF), which is described as cancer-induced cardiac cachexia. Cardiac dysfunction may reduce left ventricular systolic function in the form of heart rate, fractional shortening, cardiac output, and left ventricular mass as well as changes in left ventricular diastolic function in the form of a decrease in the E/A ratio and the E/e’ ratio and the prolongation of the isovolumetric relaxation time (IVRT). The cardiac cachectic phenotype also results in a decrease in the cross sectional area of cardiomyocytes, collagen deposition and the induction of fibrosis, the modulation of cardiac troponin I (TnI) expression, and a shift in the MHC isoform [18]. Intriguingly, a large-scale study conducted in Canada on 16,500 cancer patients who died during the period of 1993–2000 had comorbid cardiac disorders such as ischemic cardiomyopathy (12.2%), heart failure (7.5%), and conduction disorder (2%). However, certain types of cancers are more prone to develop heart failure, such as multiple myeloma (14.4%), leukemia (10.4%), lymphoma (9.7%), and lung cancer (8.5%). Whereas cancer patients with brain, oral cavity, and pharynx cancers have a much smaller (1.5–3%) chance of developing heart failure [66]. On the contrary, Roderburg et al. 2021 [67] carried out a retrospective cohort study on 100,124 heart failure patients and documented the greatest correlation with lip, oral cavity, and pharynx cancers with a hazard ratio (HR) of 2.10 and a 95% confidence interval: 1.66–2.17; *p* < 0.001, followed by respiratory organs with HR 2.1. These studies emphasize that cachexia and heart failure have mutual effects in cancer patients. The initial concept and plausible explanation for cancer-mediated cardiac abnormalities was first introduced by Burch et al. [68]. Various common mechanisms, including systemic inflammation, metabolic remodelling, angiogenesis, and the somatic mutation of epigenetic regulators contributing to both the pathophysiology of tumor growth and heart failure could explain the co-occurrence of cardiac dysfunction in cancer patients [53]. Various experiments on cachexia-inducing tumor-bearing animal model and in vitro studies have documented the frequent association of cardiac dysfunction [69] and cancer. Some in vivo and in vitro preclinical studies are presented in Table 1, and clinical studies are presented in Table 2. However, in order to avoid repetition, studies involving cardiac changes observed following various therapies such as exercise training are not included in these tables.

The augmented expression of the matrix metallo proteinases (MMP) might be responsible for the matrix remodelling and cardiac fibrosis leading to the cardiovascular disorder observed in tumor-bearing animals [70]. Altered cardiac structure and function caused by the decreased expression of contractile protein, Troponin I, and myosin heavy chain α (MHC-α), the determinant of power generation in cardiac muscle as well as the elevated expression of inflammatory marker IL-6 and its receptor might have contributed to some extent [71]. A significant shift in the MHC isoform from its adult-α form to its embryonic-β form leading to cardiomyopathy was also observed by Kelm et al. [18] in a tumor-bearing mice model.

Sex-based differences were observed in cardiac atrophy induced by colon-26 adenocarcinoma, being more severe in male compared to in female mice [30]. This difference could be due to the existence of estrogen signaling as well as the higher protein kinase activity in female myocytes that are required for the maintenance of cardiac structure and function, which could be responsible for the comparatively less severe cancer-induced cardiac effects observed in female animal [72]. Moreover, a significant reduction in aortic pressure and aortic velocity was observed in male mice, while no such change was involved in female mice. The myocyte size also decreased more in the hearts of male mice compared to in female mice. However, autophagy played a more crucial role in proteolysis during cardiac atrophy rather than the ubiquitin proteasome system (UPS) [30].

In a six-week-old female NSG immunodeficient mice model intraperitonially inoculated with A2780 ovarian cancer cells, Kakar and his group [18] recently documented that ovarian cancer could induce morphological and functional alterations in the hearts of tumor-bearing animals compared to in the tumor-free control animals. A decrease in the absolute body weight along with a bilateral significant increase in the size of the ovaries was associated with a significant reduction in the cross-sectional area of the cardiomyocytes. Other cardiac abnormalities were significant (*p* < 0.0001) reductions in heart rate along with arrythmia, left ventricular fractional shortening (35.0 ± 3.2% vs. 58.0 ± 2.2%), and LV mass (55.6 ± 2.3 mg vs. 91.8 ± 5.5 mg) as well as diastolic dysfunction measured by E/A (0.45 ± 0.12 vs. 2.17 ± 0.05) and E/e’ (−18.6 ± 0.6 vs. −9.5 ± 0.65). Cardiac changes were associated with an increase in circulating AngII levels (2.7 ± 0.3 ng/mL vs. 1.00 ± 0.34 ng/mL, *p* < 0.0001) and in angiotensin II receptor type 2 expression (AT2R 62.89 ± 21.13-fold, *p* < 0.0001) as well as the over expression of proinflammatory cytokines (TNF-α, IL-6, MIP-2 (the mouse ortholog of human IL-8) and IFNγ in the cardiomyocytes of the ovarian cancer-generated mice compared to in the cancer-free mice. The significant switch in the MHC isoform from the α to the β state as well as the generation of an exorbitant amount of connective tissue resulted in fibrotic changes in the cardiac muscles, which substantiated the manifestations of cardiac dysfunction in tumor-bearing mice, suggesting the development of ovarian cancer-induced cardiac dysfunction (cachexia). Zimmers and her colleagues also documented that ovarian cancer could induce losses in muscle as well as in bone mass in ES-2 human ovarian cancer cells inoculated into a NOD SCID gamma mouse model [73].

**Table 1:** Evidence from in vivo and in vitro experiments on cancer-induced cardiac cachexia.

S.No	Experimental Model	Methods/Tools Used	Major Outcome	References
1.	10-week-old female CD2F1 (BALB/c × DBA/2 F1) mice were injected with C26 cell line	Western blot analysis, quantitative real time PCR, picrosirius red quantification, hydroxyproline assay	In LV: Significant elevation of mRNA of TIMP 1, MMP-2, MMP-3, and MMP-14; no change in TIMP2 mRNA or in the protein levels of MMP-2, MMP-3, MMP-9, and MMP-14; and TIMP 2 elevated	[70]
2.	Rat AH-130 hepatoma CC model	NMR spectroscopy, infrared monitoring system to monitor movement, echocardiography, invasive hemodynamic assessment, ECG, Luminex-200 system, RIA, ELISA	Decrease in LV mass, heart weight, LVEDD, LVEF, LVFS, LVESP, LVEDP. Maximum response observed 13 days after inoculation	[69]
3.	Colon -26 adenocarcinoma cell line-injected male CD2F1 mice	Transthoracic echocardiography, transmission electron microscopy, RT-PCR, and Western blotting	Decreased heart rate and fractional shortening, disrupted cardiac muscle morphology, increased heart muscle fibrosis, decreased expression of TnI and MHCα, and increased expression of IL-6 and IL-6 R	[71]
4.	Colon -26 adenocarcinoma cell line-injected male CD2F1 8-week-old male and female mice	Echocardiography, electron microscopy, RT-PCR and Western blotting, ubiquitin conjugation assay	Pronounced cardiac atrophy in male mice with reductions in myocyte size and sarcomere protein and autophagy activation	[30]
5.	Ectopic mouse model: 9–10-week-old male Balb/c, C57BL6/N, and Fox Chase SCID inoculated with adenocarcinoma cells; C26 mouse, MC38 mouse, SW480 mouse, and orthotopic mouse model (PDAC): 10 week old male C57BL6/J, genetic mouse model (APC delta 580 mice); diabetic mice: 9–11 week old female ob/ob and db/db C26; MC38 and HEK293A cell culture	Echocardiography and pressure–volume loop measurement, echo magnetic resonance imaging, ELISA, gene expression analysis	Decrease in heart weight, volume, myocyte diameter, and deterioration of cardiac function in C26 transplanted and APC mutant cachectic animals and elevated ataxin-10 levels in C26 and SW480 and no fibrosis in the cachectic animals	[65]
6.	Male and female 7-week-old C57BL/J6 mice inoculated with Lewis lung carcinoma (LLC1) cells	Echocardiography, fractional protein synthesis rate, mRNA sequencing, transmission electron microscopy of mitochondria, immunoblot assay, histology	Decrease in IVS thickness, EF, %FS, fractional protein synthesis, cardiac mitochondrial oxygen consumption rate, and Complex II, III, IV and V proteins and in Apl, Aplr, N-Myc, Egr1 and Sox9 mRNAs; increase in LV ID (S + D), (AR), and (FF); increased Gadd45b; no cardiac fibrosis	[74]
7.	Eight-week-old, male C57BL6/J mice inoculated with Lewis lung carcinoma cells and H9c2 ventricular cardiomyocytes culture	Two-photon excitation fluorescence, immunoblot analysis, gas chromatography–mass spectrometry, bioenergetic flux analysis	Lower heart weight lower (10%); low optical redox ratio (15%); increased COX-IV (50%); decreased VDAC (50%); lower Cytb (mt-DNA) (30%); and lower GPx-3 and GPx-7 (~50%)	[33]
8.	Six-week-old female mice inoculated with A2780 ovarian cancer cells.	Echocardiography, histology with haematoxylin and eosin staining and Masson’s trichrome staining, qPCR for RNA quantification, and ELISA	Decreased heart rate, FS%, CO, LV mass, E/A ratio, E/e’, CSA of cardiomyocytes, cTnI, shiftin MHCα to β and increase in AngII, AT1aR mRNA, IL-6, TNFα, MIP-2, and IFNγ	[18]

LV: left ventricle, MMP: matrix metalloproteinases, TIMP: tissue inhibitors of metalloproteinases, LVEDD: LV end-diastolic diameter, LVESP: LV endsystolic pressure, LVEDP: LV end-diastolic pressure, LVEF: LV ejection fraction, and LVFS: LV fractional shortening, CC: cancer cachexia, MHC: myosin heavy chain, COX-IV: cytochrome-C oxidase subunit 4 VDAC: voltage dependent anion channel, cytb mt-DNA: mitochondrial DNA-encoded cytochrome b, GPX: glutathione peroxidase; gadd45b: mRNA for growth arrest and DNA-damage-inducible, β; Apl: apelin, Apl r: apelin receptor; AR: cardiac mitochondrial aspect ratio; FF: form factor; L VID(S + D): LV internal dimensions in systole and diastole, ECG: electrocardiography, RIA: radio immune assay, ELISA: enzyme-linked immunosorbent assay, CO: cardiac output, CSA: cross-sectional area, cTnI: cardiac troponin I, AngII: angiotensin II, AT1R: angiotensin II type 1 receptor, MIP-2: mouse ortholog of human IL-8.

The discrepancies in the findings with respect to cardiac fibrosis among various studies [65,70] might be due to the different C26 subcloned animal models used in these studies, different degrees of cachexia at the time of sacrifice, and different tumor induction techniques used in animal models (subcutaneous vs. intraperitoneal), which give rise to different tumor microenvironments as well as different amounts of AngII released into circulation by the tumor. 

Barkhudaryan et al. [2] documented that loss of body weight was significantly associated with a decrease in heart weight in autopsies of lung, gastrointestinal, and pancreatic cancer patients. A prospective study was conducted on 50 colorectal cancer (CRC) patients and 51 chronic heart failure patients and 51 healthy individuals to observe if there were any differences in the cardiovascular disturbance trends among these three groups. This was carried out by measuring their exercise ability via spiroergometry, cardiac function via echo cardiography, and heart rate variability (HRV) via 24 h echocardiography monitoring as well as a related biochemical analysis. This study documented that CRC patients had a significantly impaired exercise capacity as well as indicators of cardiovascular dysfunction, including abnormal endothelial functions [75]. A case–control study conducted on 75 patients with a pelvic mass (25 untreated ovarian cancer patients, 25 endometriosis cases, and 25 with benign tumors) showed significantly higher levels of conventional and highly sensitive cardiac troponin I (cTnI), indicating that the heart is involved in gynaecological cancer cases [76]. One study conducted both on an experimental model (AH-130 hepatoma rat model) and a cadaveric heart and the plasma level of some cancer patients documented that both the structure ((left ventricular) LV mass, presence of cardiac fibrosis, left ventricular wall thickness (LVWT)) and function of the heart progressively deteriorated with the progression of cancer [69]. In a prospective study during follow up with cancer patients, Pavo et al. [77] demonstrated that myocardial damage indicated the augmented expression of cardiovascular functional peptides and morphological markers might be responsible for disease progression and lead to increased mortality in cancer patients [77]. A multicomponent cardiovascular resonance imaging study revealed that cancer itself could modulate the morphology and function of the heart (chamber volume, ejection fraction and native T1 mapping) in naïve breast cancer or lymphoma patients [78]. A study based on the data collected from 7.5 million cancer patients from the National Cancer Institute’s Surveillance, Epidemiology, and End Results (SEER) program revealed that cancer patients are more prone to develop fatal heart disease with a standardized mortality ratio (SMR) of 2.4. Males showed a higher propensity compared to females, with a hazard ratio of 1.48 (CI 1.47–1.5%) [79]. Retrospective studies conducted by Tadic et al. [80] documented that both left ventricular and right longitudinal global stain were significantly reduced in cancer patients compared to in controls [80,81]. Later, these investigators also reported a significant left atrial reservoir and conduit function deterioration in patients with solid cancers [82].

**Table 2:** Clinical studies on evidence of cancer-induced cardiac cachexia.

S. No.	Type of Cancer (Sample Size)	Study Design/Tools Used	Outcome in Cancer Patients	References
1.	Lung cancer (58), pancreatic cancer (60), GI cancer (59)	Retrospective study on deceased cancer patients	Significantly low BMI and HW in cachectic patients, LVWT and RVWT same in both cachectic and non-cachectic patients	[2]
2.	Colorectal cancer (50)	Prospective study by 2D echo, Holter ECG, and treadmill exercise tests and biomarker analysis	Significantly reduced LVEF and HRV (SDNN, SDANN, SDNN index, VLF, and LF) parameters; significantly higher hsTnT	[75]
3.	Ovarian cancer (25)	Case–control study, highly sensitive troponin immunoassay	Significantly high hsTnI and conventional TnI	[76]
4.	Non-small cell lung or colorectal cancer patients	One set: human cadaver heart weights and wall thickness measurement Second set: plasma level of biochemical parameters from cancer patients	Reduction in heart weight (25.6%) and LVWT (12.1%); cardiac fibrosis+, increased plasma Aaldosterone, BNP, and renin levels in cancer patients	[69]
5.	Total: 555 (breast: 146, lung: 61, GI: 67, myelodysplastic: 68, myeloproliferative: 99, brain:23, ENT: 33)	Prospective study with follow up with a median of 25 months; venous blood analysis of cardiovascular functional peptide and morphological markers	(CRP), haptoglobin, fibronectin, (SAA), interleukin 6 (IL-6), hsTnT and NT-proBNP, (MR-proANP), (MR-proADM), CT-proET-1 and stable copeptin: all were elevated	[77]
6.	Chemotherapy naïve breast cancer or lymphoma (381)	Part of prospective study CAPRI (NCT04367220), cardiovascular magnetic resonance imaging	Smaller chamber volume, higher global strain amplitude, increased septal and lateral wall native T1 mapping among patients with cancer	[78]
7.	122 Solid tumors (gynaecological: 20, breast: 19, GI: 51, sarcoma 13, lungs; 19)	Retrospective study/Echo cardiography with 2D strain analysis of the left ventricle	No difference in LV diameter, wall thickness; siginificant decrease in LVEF and in LV longitudinal, circumferential, and radial strain	[80]
8.	101 Chemo- and radiotherapy in naïve cancer patients	Retrospective study/echo cardiography with 2D strain analysis of the right ventricle	Significant decrease in global RV longitudinal strain	[81]
9.	92 Chemo- and radiotherapy in naïve solid cancer patients	Retrospective study/echo cardiography with 2D strain analysis of the left atrium	Significant increase in LAVmin/BSA and LAVpre-a/BSA: decrease in LA Tot EF (%), LA Pass EF (%); decrease in longitudinal as well as systolic and early diastolic strain	[82]

HW: heart weight, GI: gastro intestinal, BMI: body mass index, LVWT: left ventricular wall thickness, RVWT: right ventricular wall thickness, SDNN index: mean of the standard deviation of normal RR intervals every 5 min, LVEF: left ventricular ejection fraction, SDANN: standard deviation of the average RR intervals for each 5 min segment of a 24 h heart rate variability recording, VLF: very low frequency, LF: low frequency, hsTnT: high-sensitivity troponin T, CAPRI: cardiotoxicity prevention research initiative, CRP: C reactive protein, SAA: serum amyloid A, MR –pro ANP: mid-regional pro-ANP, MR-Pro ADM: mid-regional pro-ADM, LA Tot EF (%), LA Pass EF (%): total and passive left atrial ejection fraction, BSA: body surface area. SD: standard deviation.

## 7. Synergistic Effect of Chemotherapy on Cancer-Induced Cardiac Cachexia (Dysfunctions)

The concept of chemotherapy-mediated cardiac dysfunction was first documented by Von Hoff et al. in 1979 [83]. This new emerging branch of cardio-oncology was developed to sort out the side effects generated by the chemotherapies that are administered for management of a specific cancer. In addition to the signs and symptoms developed by the tumor–host interactions mediated by the tumor-derived factors, chemotherapies might have detrimental effects on the cardiovascular system, which makes patient management quite challenging since this may further increase the risk of cachexia and deteriorate the existing condition rather than providing relief to cancer patients. Some of cardiotoxic chemotherapies include anthracycline (doxorubicin, daunorubicin), human epidermal growth factor receptor 2 inhibitors (HER-2) (trastuzumab, pertuzumab), alkylating agents (cyclophosphamide, busulfan), taxane (paclitaxel), platinum-based therapies (carboplatin, cisplatin), fluoropyrimidine (5-fluorouracil), tyrosine kinase inhibitors (imatinib) VEGF inhibitors (bevacizumab), immune check point inhibitors (pembrolizumab), and proteasome inhibitors (bortezomib) [84]. 

In a preclinical study, healthy 8-week-old CD2F1 male mice treated with multi kinase inhibitors (MKI) (regorafenib (30 mg/kg/day) and sorafenib (60 mg/kg/day)) for 6 weeks showed structural and functional perturbations in the heart, suggesting cardiac toxicity that was characterized by significant reductions in heart weight, stroke volume, left ventricular mass, and the left ventricular inner wall diameter (LVID). This cardiotoxicity was mediated by the enhanced phosphorylation of AKT and its downstream effectors mTOR, P70S6K, and GSK3β as well as MEK and its downstream mediator ERK1/2. Moreover, MKIs also caused the downregulation of the mitochondrial homeostasis regulatory protein OPA1 and cytochrome C. Interestingly, proteolysis marker, the ubiquitinated protein, was downregulated, and the antiapoptotic marker Bcl2 was upregulated by the MKIs, while the autophagy markers LC3 and Beclin1 remained unchanged in the cardiac muscles of the experimental animals [85]. Other experimental studies related to chemotherapy-induced cardiac effects are listed in Table 3.

Left ventricular diastolic dysfunction is common after anthracycline administration. Tamoxifen-induced cardiotoxicity was also assessed by documenting increasing trends in the cardiac-specific biochemical markers [59]. Trastuzumab, a humanized monoclonal antibody, induces cardiotoxicity via the inhibition of autophagy through the altered regulation of HER2 signaling, leading to an enhanced accumulation of ROS in primary human cardiomyocytes [99]. A retrospective observational study conducted on 55 patients (39 males and 16 females) suffering from gastrointestinal stromal tumors (GIST) and chronic myeloid leukemia (CML) reported that a newer generation of tyrosine kinase inhibitors (NTKI) such as nilotinib, ponatinib, and dasatinib were responsible for the more frequent onset of toxic cardiovascular events compared to older generation TKI (OTKI) (Imatinib). Cardiac status was evaluated by electrocardiography, echocardiography, and carotid ultrasound scans. NTKI significantly reduced left ventricular global longitudinal strain (GLS) and diastolic function in the form of the septal E’ wave while significantly increasing the E/e’ ratio, the incidence of preclinical atherosclerosis, and arterial stiffness compared to OTKI [100]. Hence, in order to identify these high-risk individuals, the American College of Cardiology and American Heart Association (ACC and AHA) recommends the pre-treatment measurement of left ventricular function as a part of standard care protocols for the management of all cancer patients, with special preference for early breast cancer patients [101]. Some clinical studies related to chemotherapy-induced cardiac effects are listed in Table 4.

In brief, both preclinical and clinical studies have documented that chemotherapy, which is the treatment of choice for cancer patients, could not only induce skeletal muscle wasting, but it may also induce cardiac dysfunction in the form of reductions in LVEF, elevated cardiac troponin I, and decreased glutathione peroxidase. Moreover, newer generation TKI are more cardiotoxic than the older generation ones. 

## 8. Therapeutical Approach for Cardiac Cachexia

Because of its complex metabolic nature and the involvement of multiple factor-mediated signaling cascades, the application of a single therapy may not be a successful strategy to control the phenotype related to cancer-induced cardiac cachexia. Therefore, we have covered two broad approaches: non-pharmacological and pharmacological approaches. 

### 8.1. Nonpharmacological Therapy

Several reports indicate that deficiency of vitamins and other micronutrients may contribute to the pathophysiology of various cardiovascular disorders [107]. According to the definition of cancer cachexia, the weight loss that is induced by this condition cannot be reversed by the nutritional supplementation. However, A randomized controlled trial NCT01172314 conducted on 13 stage III and IV non-small-cell lung cancer patients revealed that dietary essential amino acid intake could induce anabolic response in advanced cancer patients irrespective of inflammatory status, muscle mass, and disease trend, and thus, personalized nutritional supplementation could be recommended as a novel strategy to prevent the development of cachexia [108]. Hence a large- scale controlled trial is warranted to clarify the benefits of introducing customized nutritional therapy for the management of patients with cancer cachexia. 

Cumulating evidence has revealed that regular endurance exercise could induce the molecular and metabolic remodeling of heart via the activation of cardiac myocytes gene expression ((CBP/p300-interactive transactivator with ED-rich carboxy-terminal domain-4 (Cited4)), resulting in an increase in cardiac mass and contractile function and an eventual decrease in the risk of mortality [109]. Parry and Hayward [110] conducted a wheel run endurance exercise test for 6 weeks on 344 Fisher rats that had been inoculated with MatBIII tumor cells and reported that exercise showed cardioprotective effects. This cardio protection was probably mediated by the downregulation of the autophagic pathway, which is consistent with the decrease in LC3-II expression and the increase in the expression of p62 markers. Moreover, the exercise ameliorated the left ventricular developed pressure (LVDP) and rate of pressure development (+dP/dt) as a result of suppressing the shift of the cardiac myosin heavy chain protein (MHC) isoform from α to β [110]. Consistent with these observations, Fernandes et al. [111] observed the effects of aerobic exercise training (AET) on a C26 tumor-bearing mouse model and reported an improvement in the exercise capacity in these mice. Exercise training could not revert the cardiac atrophy completely but produced a partial effect on the size of cardiac myocytes and the interventricular septal wall thickness and resulted in a partial improvement in the left ventricular ejection fraction. AET also mitigated the magnitude of inflammation and thus the volume of the cardiac necrotic area. It also attenuated the cardiac fibrosis by decreasing the accumulation of both the total as well as the type I and III collagen fibrils in heart of tumor-bearing mice via the downregulation of TGFβ gene expression. AET also restored mitochondrial homeostasis through the augmentation of mitochondrial complex IV protein expression and the modulation of autophagy-related protein LAMP2 and ATG1 expression [111]. An *N*-butyl-*N*-(4-hydroxybutyl)-nitrosamine-induced urothelial cancer animal model subjected to treadmill exercise for 13 weeks revealed that endurance exercise could revert cardiac atrophy and fibrosis as well as ameliorate oxidative ability via the hyperactivation of citrate synthase and mitochondrial superoxide dismutase. In addition, this training might help cardiac regeneration through the abrogation of the cancer-mediated reduction of cardiac c-kit levels [112].

These beneficial effects of exercise have been supported by various clinical studies. MacVicar et al. [113] conducted a randomized controlled trial to observe the effects of 10 weeks of supervised cyclo-ergometry on 45 patients with breast cancer who were receiving adjuvant chemotherapy and reported that aerobic training could improve VO_2_ peak (peak oxygen consumption) by 40% compared to usual care. Later on, various studies conducted on breast cancer patients [114,115,116,117] as well as on patients with lymphoma [118], prostate cancer [119], and colorectal cancer [120] documented a significant improvement in the VO_2_ peak, which indicates cardiovascular fitness. On the contrary, Rogers et al. [121] did not observe any differences in the VO_2_ peak following 4 weeks of supervised and 8 weeks of unsupervised aerobic exercise 3–5 days per week conducted on 222 breast cancer survivors compared to usual care. Another retrospective analysis conducted on 90 patients with history of cancer who were registered in the HF-ACTION trial could not find any improvement in the VO_2_ peak; rather they, found an increase in the incidence of cardiovascular mortality in the training group, compared to the usual care group [122]. The heterogenous end point as well as inconsistency in the safety and efficacy of the prescribed exercise, even in an apparently homogenous population observed in various studies, could be due to the diversity in the mode of treatment, such as chemotherapy and or radiotherapy; pre-existing cardiovascular risk factors such as age, hypertension, dyslipidemia, obesity, smoking habits; and basal health status, such as the left ventricular ejection fraction and VO_2_ peak with respect to age and sex. Hence, it appears that a standard fixed-dose exercise protocol would not be appropriate for all cancer patients. To improve the end-point efficacy of exercise protocols, all cancer patients should be stratified according to their individual risk factors, management strategy, and baseline physiological status. To cope with cancer-induced cardiac cachexia, a targeted as well as customized exercise training prescription considering exercise type, intensity, and duration should be developed and tailored to that specific cancer patient population. 

### 8.2. Pharmacotherapy

The existing standard therapeutic strategy for cardiac cachexia includes ACE inhibitor, angiotensin II type I receptor blocker, β blockers, and diuretics. The potential role of ACE inhibitors in delaying the progression of the disease can be attributed to the improvement of endothelial function, the normalization of circulating IGF-1, and significant reductions in cardiac markers such as ANP, BNP, IL-6, and TNFα levels [64].

The pharmacotherapeutic options for combating cardiac muscle wasting are not well defined. It is a great challenge to treat muscle wasting with novel therapies that target the mediators of the molecular pathways involved in pathogenesis of cardiac cachexia in cancer patients since these have yet to receive approval. Considering the major role of inflammation in the induction of cardiac dysfunction in cancer patients, various studies were conducted to target pro-inflammatory markers such as the IL-1α antagonist (MABp1) [123], IL-1β inhibitor (Canakinumab) [124], IL1β receptor antagonist (Anakinra), TNFα antagonist (Infliximab and Etanercept) [125], and IL-6 antagonist (Clazakinumab) [126]. Out of these compounds, only the TNFα antagonist failed to provide any beneficial effects in the form of quality of life or cardiac function [127]. Selumetinib, which blocks MEK1/2-dependent ERK1/2 phosphorylation, and thalidomide, which inactivates the NFkB and TGFβ/SMAD signaling pathway, might pave the way for the restoration of cardiac function in cancer patients [27]. One nonrandomized phase II trial was conducted to observe the effects of celecoxib (COX-2 inhibitor) on patients with cancer cachexia and reported a significant improvement in body weight and quality of life and a decrease in TNF alpha [128]. Subsequently, two systematic reviews carried out by Reid et al. [129] and Solheim et al. [130] documented that NSAIDs may have a beneficial effect with respect to gains in lean body mass and improvements in quality of life in cancer cachexia. However, the authors of both review articles concluded that recommending the broad-spectrum use of NSAIDs for the treatment of patients with cancer cachexia remains inconclusive because of a lack of adequate evidence. 

Loss of appetite is a characteristic feature of cancer cachexia that reduces exercise tolerance and eventual cardiac remodelling in some advanced cancer patients. Hence, improving appetite could be a good target for preventing disease progression. Megestrol acetate (MA) (100 mg/kg/day), an orally active appetite enhancer, administered in Yoshida AH-130 ascites hepatoma rats showed a significant amelioration of the left ventricular ejection fraction (LVEF), fractional shortening (LVFS), and end systolic volume (LVESV) via the modulation of the effectors related to the autophagy pathway, such as Beclin1, LC3, and P62 in cardiac muscle, indicating the beneficial role of MA in counteracting cancer-induced cardio-myopathy [131]. Garcia et al. [132] conducted a meta-analysis that included 23 RCTs from 3428 patients with different cancers to evaluate the safety and efficacy of the progesterone analogue Megestrol acetate (MA) on anorexia–cachexia syndrome. These investigators reported that MA significantly improved appetite (with a relative risk (RR) of 2.57; 95% CI, 1.48 to 4.49), quality of life (with RR, 1.91; 95% CI, 1.02 to 3.59), and weight (with RR, 1.55; 95% CI, 1.06 to 2.2) in intervention arm compared to the control arm [132]. A three-arm trial of megestrol acetate, dexamethasone, or fluoxymesterone conducted on 496 randomly selected advanced cancer patients documented that MA was more effective compared to fluoxymesterone with respect to improvements in appetite [133]. One double-blinded placebo-controlled study conducted on 116 advanced gastro-intestinal cancer patients reported that Corticosteroid (dexamethasone) had a beneficial effect in terms of appetite improvement as well as wellbeing [134]. This was later supported by a systematic review involving five more trials [135]. Two (ROMANA 1 and ROMANA 2) placebo-controlled phase 3 randomised trials conducted on 979 cachectic advanced non-small-cell lung cancer patients reported that anamorelin, a novel ghrelin-receptor agonist, significantly increase lean body mass without having an effect on hand grip strength [136]. However, the US Food and Drug Administration (FDA) did not approve it for the treatment of cancer cachexia patients. The ASCO (American Society of Clinical Oncology) guidelines do not recommend any of the following pharmacological interventions: anamorelin, olanzapine, androgens, thalidomide, cyproheptadine, cannabinoids, and NSAIDs, for the treatment of cancer-induced cachexia patients [137]. Further studies considering both improvements in appetite as well as cardiac components are required to observe the effect of appetite stimulants on cancer-induced cardiac cachexia patients. 

Poor prognosis, unfavourable treatment protocols, and uncertainty about disease trajectory could precipitate depression in cancer patients. Studies have reported that depression might further mitigate appetite, promote refractoriness to chemotherapy, and reduce immune response and thus further deteriorate the existing condition [138]. The administration of the tandospirone (10 mg/Kg/day) 5-hydroxytryptamine 1A (5-HT1A) receptor agonist, which has antidepressant activity, was able to restore the heart weight and function in the form of LVEF, stroke volume, and LVFS in an 8-week-old male Yoshida hepatoma rat model [139]. Although the exact cardioprotective mechanism of tandospirone was not elucidated, the tandospirone-mediated improvements in appetite could have helped in the preservation of the cardiac mass and function of the rat model to some extent. However, this provides insight into possible future roads addressing the psychological component to prevent further clinical progression in cancer-mediated cardiac cachexia [139]. 

A previous study revealed that dysregulated matrix metalloproteinases (MMPs) could contribute to detrimental molecular remodelling, resulting in cardiac abnormalities. A preclinical study conducted on 10-week-old CD2F1 female mice inoculated with C26 adenocarcinoma cells revealed that the administration of minocycline, an inhibitor of MMP, could improve cardiac function by restoring the contractility of cardiac myocytes and attenuating collagen I and III expression in the cardiac muscles [140]. Simvastatin (10 mg/kg/day once daily for 14 days), the inhibitor of the production of matrix metalloproteinase-9 and the drug of choice for dyslipidaemia, which is an important risk factor for cardiovascular disorders, was able to improve cardiac output (57.0 mL/min) in young Wister Han rats that had been injected with Yoshida AH-130 hepatoma cells compared to those who had been injected with a placebo (42.4 mL/min) and thus decreased the mortality rate (HR: 0.16, 95% CI: 0.04–0.76, *p* = 0.021) in the treated animals [141]. However, in the same experimental model. Muscaritoli et al. [142] documented that simvastatin significantly reduced heart weight. Further studies are required to sort out the controversial role of simvastatin. In an experimental model of tumor-bearing Wistar rats, erythropoietin ameliorated cardiac function indirectly by improving physical performance by preventing muscle wasting and directly by improving cardiac mass, LVEF, LVFS, and stroke volume and thus improved the overall survival of the tumor-bearing experimental animals [143]. 

**Figure 5:**
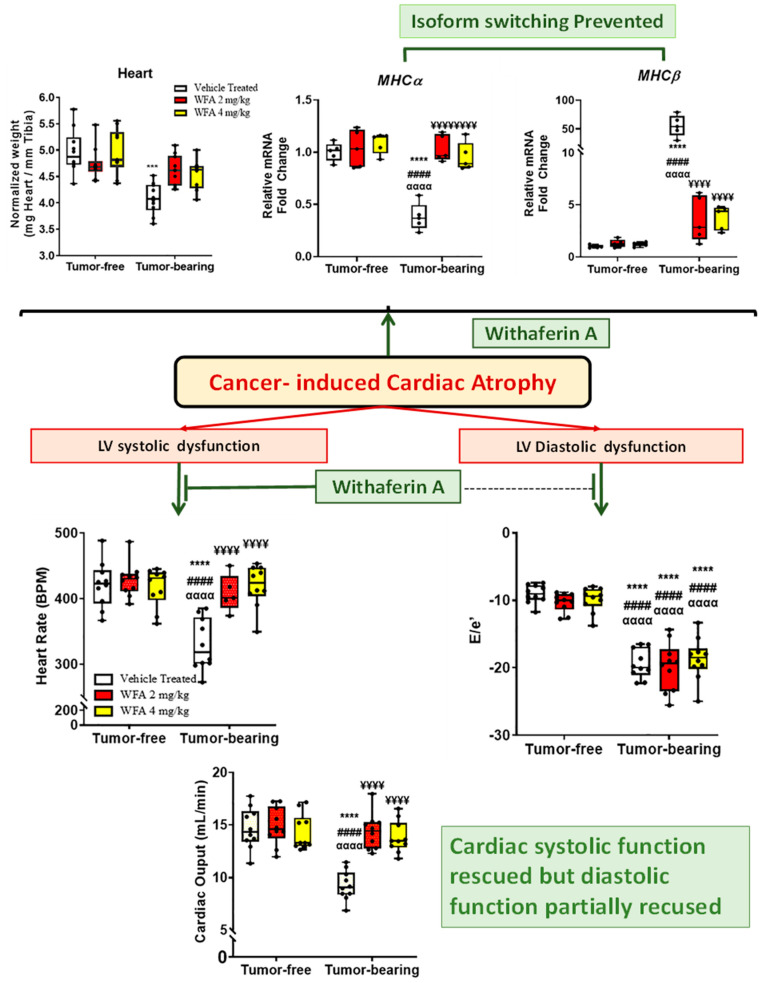
Effect of Withaferin A (WFA) on the reversal of cardiac cachexia induced by cancer. Reversal of cardiac systolic functions by Withaferin A in the form of reversal of heart weight, left ventricular heart rate, fractional shortening, cardiac output and left ventricular mass, cross-sectional area of cardiomyocytes (not shown), and partial reversal of diastolic function (E/A ratio, E/e’ ratio, IVRT: isovolumetric relaxation time), and shift in MHC isoforms) through the decrease in angiotensin II and proinflammatory cytokines levels in an NSG mice tumor model. *** *p* < 0.001; **** *p* < 0.0001 indicates a significant difference from the corresponding value of the tumor-free vehicle-treated group by two-way ANOVA followed by Tukey’s multiple comparison test, #### *p* < 0.0001 indicates a significant difference from the corresponding value of the tumor-free WFA 2 mg/kg group, αααα *p* < 0.0001 indicates a significant difference from the corresponding value of the tumor-free WFA 4 mg/kg group. ¥¥¥¥ *p* < 0.0001 indicates a significant difference from the corresponding value of the tumor-bearing vehicle-treated group. Adopted from reference [18]. Reproduced under Attribution License (CC BY 4.0).

A steroidal lactone “Withaferin A”, a purified plant extract from Ashwagandha (2 mg/Kg or 4 mg/Kg), was administered in a xenografted ovarian cancer model and demonstrated the potential to restore the cardiac cachectic phenotype, which was represented by the amelioration of left ventricular mass changes, the preservation of cardiac systolic and diastolic function (partial), the mitigation of a cancer-induced decrease in the size of the cardiac myocytes and cardiac TnI that are probably due to downregulation of the circulating proinflammatory cytokines, the TNF-α and AngII levels, and a reduction in TNF-α- and AngII -mediated MHC Isoform switching in the cardiac myocytes [18]. The effects of Withaferin A on cancer-induced cardiac cachectic phenotype are presented in Figure 5.

Doxorubicin, a commonly used chemotherapy drug for the treatment of cancer patients, is known to cause cardiotoxicity because it induces the apoptosis of cardiomyocytes that can be alleviated by glucocorticoid. In an in vitro model of H9c2 cells, glucocorticoid exhibited its cardioprotective effects through the overexpression of the glucocorticoid-induced leucine zipper (GILZ) gene in the cardiomyocytes, which inhibited the doxorubicin-induced apoptosis of cardiomyocytes via the activation of the pro-survival gene Bcl-xL [144]. Legi et al. [145] documented that aprepitant, an NK1R (a high affinity substance P receptor) antagonist, could mitigate doxorubicin-mediated cardiac dysfunction in a 6-week-old male C57BL/6 mice model, indicating the involvement of substance P and its high affinity receptor in the development of chemotherapy-induced cardiac toxicity and NK1R as a therapeutic target for the attenuation of these chemotoxicities [145]. Springer et al. [69] documented that the beta-1 selective antagonist bisoprolol 5 mg/kg/day and the aldosterone antagonist spironolactone 50 mg/kg/day abrogated the cardiac dysfunction and ultimately ameliorated the survival of AH 130 hepatoma rats, while different doses of the angiotensin-converting enzyme inhibitor imidapril (0.4 to 10 mg/kg/day) failed to show any improvement [69]. Despite the promising and encouraging results observed in experimental models, the attempt to translate it into clinical studies is still in its infancy. Therefore, large-scale clinical studies are urgently required to develop comprehensive guidelines for the prevention and care of cardiac abnormalities in cancer patients/survivors. 

In a nutshell, customized exercise and nutritional support may be tried to target patients with different cancer types and severities. Various appetisers and antidepressants have shown promising results in the improvement of cancer-induced cachexia. However, their role in cardiac cachexia needs to be thoroughly elucidated. Although some plant extracts such as Withaferin A and the MMP inhibitor Minocycline showed encouraging results in the improvement of cardiac function in an experimental model of cancer-induced cachexia, large-scale clinical studies are recommended to identify their beneficial impact in cancer patients.

## 9. Clinical Trials

Some of the clinical trials on various cardiovascular drugs, exercise, and nutritional supplements that have been registered in clinicaltrial.gov (accessed on 3 March 2022) [146] to observe their efficacy in preventing or reducing the severity of chemotherapy-induced cardiac toxicities are presented in Table 5. 

In summary, some clinical trials are registered to observe the effects of drugs and exercise on cardiac effects in cancer patients. However, a few trials have been completed that have showed that carvedilol and exercise had partially opposite effects on the cardiovascular system. Carvedilol reduced troponin levels, whereas exercise increased troponin levels. Some studies have been completed, but detailed results are unavailable. Some studies had to be terminated because of slow accrual, which may be due to a poor response from cancer patients because of their physical and mental condition. This inadequate information might hinder conclusion regarding therapeutic interventions for cardiac cachexia from being drawn. The publication of results after the completion of all ongoing trials may provide some insight into the therapeutic management of patients with cancer-induced cardiac cachexia. More clinical trials targeting the etiopathogenesis of cancer-induced cachexia might be encouraged to determine a highly specific therapy for these patients. 

The studies mentioned in Table 1, Table 2, Table 3, Table 4 and Table 5 is not exhaustive. Some frequently cited studies conveying messages relevant to their respective subtopics are included in this article. Special precaution has been taken to avoid the repetition of the same study in different tables. 

## 10. Future Prospects

As reported above, Withaferin A (WFA), which is extracted from the plant Ashwagandha (*Withania somnifera*), could ameliorate cardiac dysfunction in an experimental model of female mice with ovarian cancer by reversing systolic function, revoking the decrease in the size of the cardiac myocytes, ameliorating cardiac fibrosis, abrogating MHC isoform shifting, and significantly reducing the circulating AngII and proinflammatory cytokines [18]. Withaferin A also showed significant improvements in the skeletal muscle mass in a tumor-bearing mice model [149]. Therefore, Withaferin A could be a potential therapy for the prevention and treatment of the cardiac cachexia observed in cancer patients. Therefore, translational research is warranted to observe its clinical implications as a cost-effective and efficacious therapeutic option. 

The significant critical association between the aberrant expression of microRNA (miRNA) and cancer was first documented by Calin and colleagues [150]. Later on, He et al. [151] demonstrated the central role of exosomal miRNA-21 in the prevention of lung and pancreatic cancer cell-mediated muscle wasting through the activation of Toll-like receptor 7/8, which is expressed in muscle cells. miRNA-21 was also linked to cardiac fibrosis, which is a characteristic structural alteration that leads to the cardiac dysfunction observed in cancer patients [152]. Bei and Xiao [109] reported the cytokine-mediated downregulation of miRNA-486 in both skeletal and cardiac muscles in both in vivo and in vitro models. In addition, the involvement of miRNA1 and miRNA133 were reported to be involved in the morphological alteration of cardiac and skeletal myocytes [153]. Studies are warranted to establish the potential role of miRNAs in cancer-induced cardiac cachexia, which could serve as a therapeutic target for improving survival in cancer patients. 

Various clinical and preclinical studies [154,155] have documented a significant increase in IL-6 and lipopolysaccharide-binding protein (LBP) as well as gut barrier dysfunction resulting in the release of proinflammatory gut microbiota, giving rise to endotoxemia and a metabolic imbalance, a characteristic phenotype observed in cachectic models. A significant reduction in Lactobacillus species and an increase in Enterobacteriaceae species were found to be linked to a cachectic phenotype of a leukemia mice model, indicating the role of the gut microbiota in the development of cancer cachexia [156]. Moreover, gut microbiota-derived products such as short chain fatty acids were found to be associated with the development and progression of different cardiovascular disorders, including heart failure [157]. Thus, a better understanding of the mechanisms of how the gut microbiota are connected to cardiac dysfunction in cancer patients might be helpful in finding out a potential therapeutic target for the treatment of cancer-induced cardiac cachexia and for the eventual improvement of quality of life and overall survival. 

## 11. Conclusions

From the present review, we can conclude that cancer-induced cachexia has a significant impact on the cardiac phenotype, which includes loss of heart weight, the development of fibrosis, a decrease in the ejection fraction, and fractional shortening, resulting in cardiac dysfunction and poor survival. Tumor-derived proinflammatory cytokines, AngII, reactive oxygen species, immune modulation, metabolic imbalance, and proteolysis by autophagy and/or UPS activity are the major driving pathophysiology events in cancer-induced cardiac cachexia [18]. In addition to tumor–host interactions, chemotherapy itself may cause cardiac toxicity, lowering quality of life and increasing the risk of early death. Various clinical trials with drugs targeting proinflammatory cytokines and beta blockers such as statins and ACE inhibitors are being performed for some of disorders and show promising results. The potential therapeutic effects achieved with plant extracts such as Withaferin A in mitigating the cardiac phenotype in animal models is quite encouraging. However, in depth research is required to pinpoint the specific underlying mechanisms/pathogenesis and to sort out the discrepancies observed in various studies. Moreover, the promising findings obtained from preclinical studies need to be translated into clinical studies to find out the most efficacious and effective therapeutics for cardiac dysfunction, problems that mostly remain unaddressed in cancer patients. Therefore, collaborative research at the preclinical and clinical levels among cardiologists and oncologists would help in determining a standardized and customized protocol for the management of cancer-induced cardiac cachexia patients.

## Figures and Tables

**Table 3:** Experimental models presenting chemotherapy-mediated cardiac effects.

S.No	Name of the Drug	Experimental Model	Outcome	Reference
1	Doxorubicin (Doxo)/epirubicin (Epi), pirarubicin, daunorubicin (Dauno)	Perfusion of isolated hearts from treated (for 11 days on alternate day) and untreated 10-week-old male Sprague-Dawley rats that were sacrificed on the 12th day	Doxorubicin and daunorubicin: most toxic agents. Doxo caused a 33% reduction in [LV(dP/dt)_max_], a 29% decrease in [LV(dP/dt)_min_, and less changes were observed with Epi and dauno	[86]
2	Doxorubicin or epirubicin (3 mg/kg/day), paclitaxel or docetaxel (2.5 mg/kg/day)	Perfused isolated hearts from 10–12-week-old male Sprague Dawley rats treated ip on alternate days for 11 days and sacrificed on 12 th day	Doxo: a 20% decrease in [LV(dP/dt)_max]_ and a 33% decrease in [LV(dP/dt)_min]_; paclitaxel: No significant changes; doxo + paclitaxel: a 39% decrease in [LV(dP/dt)_max]_ and a 46% decrease [LV(dP/dt)_min_]	[87]
3	Tyrosine kinase inhibitors: gefitinib, lapatinib ditosylate, dasatinib, sorafenib tosylate, erlotinib, sunitinib, imatinib	Ventricular myocytes were isolated from 2–3-day-old Sprague Dawley rats and plated in a culture dish. On the 5th day, cells were treated with drugs, and after 3 days, cells were lysed to measure LDH activity	Lapatinib, erlotinib, gefitinib: no cardiotoxicity; imatinib, sorafenib: mild; sunitinib, dasatinib: moderate toxicity. Loest LDH activity correlated with lLapatinib, and the highest was correlated with dasatinib.	[88]
4	Regorafenib (30 mg/kg/day) or sorafenib (60 mg/kg/day)	Eight-week-old CD2F1 male mice were treated with oral drugs for 6 weeks and sacrificed on day 42	EF and FS: no change in stroke volume, heart weight, or LV mass; LVID: decreased. More severe effect observed with regorafenib. Increased phosphorylation of Akt/mTOR/P70S6K/GSK3β and MEK/ERK1/2	[85]
5	Cisplatin (7 mg/kg i. p, twice a week)	5-week-old male Nu/MRI nude mice with prostate cancer. At day 27, hearts were surgically removed and used for transmission electron microscopy analysis	Increased mitochondrial damage was seen (in terms of morphology, size, organization, and quantity)	[89]
6	Four doses of 5-FU, 150 mg/kg b.wt.	Wistar male rats weighing 170–200 gms were sacrified 2 weeks after last dose of 5-FU. BCKDH activity was assayed spectrophotometrically. The mRNA levels for E1, PPM1K, and BDK were quantified by real-time PCR	Increase in myocardial BCKDH activity state. mRNA level for BDK decreased, while mRNA levels for PPM1K Increased. This ultimately leads to the deterioration of cardiac functions.	[90]
7	Twin doses of 5-FU intraperitoneal (i.p.) injection of 25 mg/kg	Sprague Dawley rats aged 2 and 18 months had blood collected from tail vein on days 0, 7, and 14. On the 15th day, the rats were sacrificed	Weight loss and myocardial injury observed in rats. Ventricular enlargement, decrease in myocardial contractile function, and decrease in LVEF in aged rats. Cardiomyocyte apoptosis, myocardial mitochondrial damage, enhanced mitochondrial autophagy	[91]
8	THP (3 mg/kg) was injected via caudal vein once a week	SD rats (180–200 g) were sacrified at the end of the 8th week.	Decreased body weight and food intake. EF and FS decreased; LVIDd and LVIDs increased; R and T wave decreased, the S wave increased, and the QT interval was prolonged.	[92]
9	Cisplatin (2 mg/kg/day) daily by intraperitoneal injection for 1 week	Male albino rats (180–220 g) rats sacrificed on 8th day	Serum levels of CK and LDH increased. The muscle fibers showed disarrangement, discontinuities, and apoptotic nuclei (seen by electron microscopy)	[93]
10	Single dose of cisplatin (CP; 10 mg/kg)	Adult male Wistar rats (weighing 180–200 g) had blood samples collected from retro-orbital venous plexus.Rats sacrificed on 16th day	Elongation of QTc with ↑ ST height and T wave amplitude; ↑ HR. Increase in serum troponin T, LDH, and CK-MB. GSH and SOD activity reduced; caspase 12 gene expresson elevated.	[94]
11	CYP (200 mg/Kg, i.p.) as a single dose.	Male Wister rats (150 ± 20 g), aged 15–17-weeks old nlood collected from orbital sinus puncture 3 days after CYP	Cardiac Tn-I protein and LDH enzyme and serum levels of IL-1ß, IL-6, and TNF-a increased; wide-spread swelling and granular and vacuolar degeneration as well as myocardial separation with intramuscular edema.	[95]
12	Single IP injection of CYP (200 mg/kg)	Wistar albino rats (7-week-old) weighing 160–180 g were sacrficed on the 11th day, and blood was collected from abdominal aorta	Body weight loss with increased heart weight to body weight ratio; increased plasma CK, LDH, AST, and cTn I levels; enhanced apoptotic signaling; upregulated PARP1 and p53 in cardiac tissue	[96]
13	Single IP injection of CYP (200 mg/kg)	Male Wistar albino rats On the 11th day, the were rats sacrificed	Increased serum LDH, CK-MB, and troponin; decreased soluble α Klotho protein and evidence of histopathological lesions in cardiac tissues; decreased gene expression of ALDH2, Klotho protein, mTOR, IGF, AKT, AMPK, and BCL2; and increased expression of BAX and caspase-8.	[97]
14	Single intraperitoneal dose of 20 mg/kg MTX	Male Wistar rats weighing 180–210 g, and rats were sacrified on the 6th day	Fragmented necrotic muscle Fibers, apoptotic morphology with hyperacidophilic cytoplasm, nuclear pyknosis, and nuclear fragmentation. Hemorrhage and congestion, increase in cardiac NOX-2, MDA, and NO levels, and decrease in the level of GSH and in SOD. Increased TNF -α and IL-6 levels.	[98]

Left ventricular-developed pressure (LVDP) and the maximal and minimal first derivatives of LV systolic pressure as a function of time: [LV(dP/dt)_max_], heart contractions and [LV(dP/dt)_min_], heart relaxation; ip: intraperitoneal, EF: ejection fraction, FS: fractional shortening, LV: left ventricle. LVID: left ventricular inner wall diameter, 5-FU: 5-fluorouracil, BCKDH: branched-chain α-keto acid dehydrogenase complex, PPM1K: protein phosphatase, Mg^2+^/Mn^2+^-dependent 1K; LVEF: left ventricular ejection fraction, LVID-s: left ventricular internal dimension in systole, DOX: doxorubicin, SD rats: Sprague Dawley rats; THP: pirarubicin; GSH: lutathione; CYP: cyclophosphamide, cTn I: cardiac troponin I, MTX: methotrexate; SOD: superoxide dismutase.

**Table 4:** Clinical studies revealing chemotherapy-induced cardiovascular adverse events.

Serial No	Name of Drug	Study Design	Significant Outcome	References
1.	Anthracycline (doxorubicin or epirubicin)	Clinical study with 140 prospectively recruited patients assessed by transthoracic echocardiogram within 7 days post-treatment	LVEF, GLS, and global circumferential straindecreased; LVESVincreased; no changes were observed in LVEDV	[102]
2.	Anthracycline or anthracycline plus trastuzumab	Analytical, observational prospective cohort study of 100 breast cancer patients,; echo cardiogram and biochemical markers measured during 4 visits (pre-treatment, immediately after treatment, and 3- and 9-months post treatment)	Significant decrease in LVEF, increase in hsTNT, NTproBNP, and FABP	[103]
3.	Tamoxifen	Prospective observational study of 30 breast cancer patients; cardiac and oxidative stress markers measured before and 6- and 12-months post treatment	Elevated cTnI and AOPP and decreased GPx	[59]
4.	Pertuzumab following trastuzumab	Two Breast cancer patients	Left ventricular dysfunction	[104]
5.	Pertuzumab	Open-label, phase II, multicenter, randomized study, 78HER2 negative metastatic breast cancer patients	8 Patients had decrease in LVEF	[105]
6.	ICI (pembrolizumab, ipilimumab, atezolizumab, avelumab)	Retrospective and prospective study involving 8 centers and 35 patients with ICI-associated myocarditis and 105 ICI-treated patients without myocarditis followed up with for 102 days	50% Patients on myocarditis developed MACE cardiovascular death, cardiogenic shock, and cardiac arrest	[106]
7.	Newer generation tyrosine kinase inhibitors (NTKI), such as nilotinib, ponatinib, dasatinib, and older generation TKI (imatinib)	55 patients with GIST and CML; evaluation by ECG, echo, and arterial scans	More frequent cardiovascular dysfunction with newer generation of TKI	[100]

LVEF: LV ejection fraction, GLS: global longitudinal strain, LVEDV: left ventricular end diastolic volume; LVESV: left ventricular end systolic volume, H-FABP: heart-type fatty acid binding protein, cTNI: cardiac troponin I, AOPP: advanced oxidation protein products, and GPx: glutathione peroxidase. MACE: major adverse cardiac events, GIST: gastrointestinal stromal tumor, CML: chronic myeloid leukemia.

**Table 5:** Clinical trials on mitigating the cardiotoxicity induced by chemotherapy.

Trial Identifier	Estimated No of Participants	Status/Phase/Results (If)	Primary Outcome	Conditions	Interventions
NCT00292526	114	Completed/4/Increase in cTnI following HDC predicts the suppression of LVEF [147]	Incidence of chemotherapy-induced cardiotoxicity	Cardiotoxicity	Enalapril
NCT03389724	200	Recruiting/3	The effect of ACE-I in preventing chemotherapy-related cardiotoxicity measuring in troponin I levels and cardiac imaging	Cardiotoxicit, AML in children	Capoten
NCT01724450	200	Completed/3/No effect on incidence of LVEF reduction, significant decrease in troponin levels and diastolic dysfunction [148]	Prevention of systolic dysfunction in patients receiving anthracycline	Breast cancer, heart failure	Carvedilol
NCT04023110	110	Recruiting/1	To measure the left ventricular ejection fraction (LVEF)	Cardiotoxicity, breast cancer	Carvedilol
NCT03650205	160	Recruiting/not applicable	Reduction in global longitudinal strain of at least 10% (GLS)	Heart failure, chemotherapy	Ivabradine
NCT02943590	300	Active/2	To determine if statins preserve the LVEF at 12 months	Heart failure	Atorvastatin
NCT03186404	112	Recruiting/2	To compare the cardiac MRI measured LVEF between placebo and statin group	Cancer, heart failure, cardiotoxicity	Atorvastatin
NCT03949634	272	Unknown/3	Congestive heart failure with clinical symptoms or no symptoms but an abnormal LVEF	Early breast cancer	Cyclophosphamide, pegylated liposomal doxorubicin
NCT03934905	70	Not yet recruiting/1 and 2	Change in cardiac function (by 2D echo) after DOX therapy with or without sulforaphane	Anthracycline-related cardiotoxicity in breast cancer	Sulforaphane (nutritional supplement)
NCT02796365	29	Completed/not available	Left ventricular strain by spectral Doppler	Doxorubicin-induced cardiomyopaty, breast cancer, gastric cancer, leukaemia	Exercise
NCT02006979	27	Completed/1/ Exercise reduced LV twist and NTproBNP and increased cTropT and GLS in the intervention group compared to control	Global longitudinal strain by 2D speckle tracking echocardiography	Breast cancer	Exercise < 24 h prior to each cycle of anthracyclines
NCT02472353	30	Terminated did not meet target accrual/2 Results available	Whether the addition of metformin will decrease the incidence of change in left ventricle ejection fraction	Breast cancer	Metformin, doxorubicin

cTnI: Cardiac troponin I, LVEF: left ventricular ejection fraction, GLS: global longitudinal strain, cTrop T: cardiac troponin T.

## References

[1] Sung H., Ferlay J., Siegel R.L., Laversanne M., Soerjomataram I., Jemal A., Bray F. (2021). Global cancer statistics 2020: GLOBOCAN estimates of incidence and mortality worldwide for 36 cancers in 185 countries. CA Cancer J. Clin..

[2] Barkhudaryan A., Scherbakov N., Springer J., Doehner W. (2017). Cardiac muscle wasting in individuals with cancer cachexia. ESC Heart Fail..

[3] Aoyagi T., Terracina K.P., Raza A., Matsubara H., Takabe K. (2015). Cancer cachexia, mechanism and treatment. World J. Gastrointest. Oncol..

[4] Fearon K., Strasser F., Anker S.D., Bosaeus I., Bruera E., Fainsinger R.L., Jatoi A., Loprinzi C., MacDonald N., Mantovani G. (2011). Definition and classification of cancer cachexia: An international consensus. Lancet Oncol..

[5] Schmidt S.F., Rohm M., Herzig S., Diaz M.B. (2018). Cancer cachexia: More than skeletal muscle wasting. Trends Cancer.

[6] Dahlman I., Mejhert N., Linder K., Agustsson T., Mutch D., Kulyte A., Isaksson B., Permert J., Petrovic N., Nedergaard J. (2010). Adipose tissue pathways involved in weight loss of cancer cachexia. Br. J. Cancer.

[7] Fouladiun M., Körner U., Bosaeus I., Daneryd P., Hyltander A., Lundholm K.G. (2005). Body composition and time course changes in regional distribution of fat and lean tissue in unselected cancer patients on palliative care—Correlations with food intake, metabolism, exercise capacity, and hormones. Cancer Interdiscip. Int. J. Am. Cancer Soc..

[8] Peyta L., Jarnouen K., Pinault M., Coulouarn C., Guimaraes C., Goupille C., de Barros J.-P.P., Chevalier S., Dumas J.-F., Maillot F. (2015). Regulation of hepatic cardiolipin metabolism by TNFα: Implication in cancer cachexia. Biochim. Biophys. Acta Mol. Cell Biol. Lipids.

[9] Goncalves M.D., Hwang S.-K., Pauli C., Murphy C.J., Cheng Z., Hopkins B.D., Wu D., Loughran R.M., Emerling B.M., Zhang G. (2018). Fenofibrate prevents skeletal muscle loss in mice with lung cancer. Proc. Natl. Acad. Sci. USA.

[10] Fearon K.C., Barber M.D., Falconer J., McMillan D.C., Ross J.A., Preston T. (1999). Pancreatic cancer as a model: Inflammatory mediators, acute-phase response, and cancer cachexia. World J. Surg..

[11] Holroyde C.P., Skutches C.L., Boden G., Reichard G.A. (1984). Glucose metabolism in cachectic patients with colorectal cancer. Cancer Res..

[12] Kokot F., Ficek R. (1999). Effects of neuropeptide Y on appetite. Miner. Electrolyte Metab..

[13] Argilés J.M., Stemmler B., López-Soriano F.J., Busquets S. (2015). Nonmuscle tissues contribution to cancer cachexia. Mediat. Inflamm..

[14] Li Q., Kumar R., Underwood K., O’Connor A.E., Loveland K.L., Seehra J.S., Matzuk M.M. (2007). Prevention of cachexia-like syndrome development and reduction of tumor progression in inhibin-deficient mice following administration of a chimeric activin receptor type II-murine Fc protein. Mol. Hum. Reprod..

[15] Reddel C., Allen J., Ehteda A., Taylor R., Chen V., Curnow J., Kritharides L., Robertson G. (2017). Increased thrombin generation in a mouse model of cancer cachexia is partially interleukin-6 dependent. J. Thromb. Haemost..

[16] Savarese G., Lund L.H. (2017). Global public health burden of heart failure. Card. Fail. Rev..

[17] Evans W.J., Morley J.E., Argilés J., Bales C., Baracos V., Guttridge D., Jatoi A., Kalantar-Zadeh K., Lochs H., Mantovani G. (2008). Cachexia: A new definition. Clin. Nutr..

[18] Kelm N.Q., Straughn A.R., Kakar S.S. (2020). Withaferin A attenuates ovarian cancer-induced cardiac cachexia. PLoS ONE.

[19] Lena A., Ebner N., Anker M.S. (2019). Cardiac cachexia. Eur. Heart J. Suppl..

[20] Martins T., Vitorino R., Moreira-Gonçalves D., Amado F., Duarte J.A., Ferreira R. (2014). Recent insights on the molecular mechanisms and therapeutic approaches for cardiac cachexia. Clin. Biochem..

[21] Aukrust P., Ueland T., Lien E., Bendtzen K., Müller F., Andreassen A.K., Nordøy I., Aass H., Espevik T., Simonsen S. (1999). Cytokine network in congestive heart failure secondary to ischemic or idiopathic dilated cardiomyopathy. Am. J. Cardiol..

[22] Anker S.D. (2002). Imbalance of catabolic and anabolic pathways in chronic heart failure: Implications for the treatment of cardiac cachexia. Scand. J. Nutr..

[23] Glickman M.H., Ciechanover A. (2002). The ubiquitin-proteasome proteolytic pathway: Destruction for the sake of construction. Physiol. Rev..

[24] Von Haehling S., Jankowska E.A., Anker S.D. (2004). Tumour necrosis factor-α and the failing heart. Basic Res. Cardiol..

[25] Gulick T., Chung M.K., Pieper S.J., Lange L.G., Schreiner G.F. (1989). Interleukin 1 and tumor necrosis factor inhibit cardiac myocyte beta-adrenergic responsiveness. Proc. Natl. Acad. Sci. USA.

[26] Anker S.D., Von Haehling S. (2004). Inflammatory mediators in chronic heart failure: An overview. Heart.

[27] Ausoni S., Calamelli S., Saccà S., Azzarello G. (2020). How progressive cancer endangers the heart: An intriguing and underestimated problem. Cancer Metastasis Rev..

[28] von Haehling S., Doehner W., Anker S.D. (2007). Nutrition, metabolism, and the complex pathophysiology of cachexia in chronic heart failure. Cardiovasc. Res..

[29] Schakman O., Gilson H., Thissen J.-P. (2008). Mechanisms of glucocorticoid-induced myopathy. J. Endocrinol..

[30] Cosper P.F., Leinwand L.A. (2011). Cancer causes cardiac atrophy and autophagy in a sexually dimorphic manner. Cancer Res..

[31] Willis M.S., Rojas M., Li L., Selzman C.H., Tang R.-H., Stansfield W.E., Rodriguez J.E., Glass D.J., Patterson C. (2009). Muscle ring finger 1 mediates cardiac atrophy in vivo. Am. J. Physiol. Heart Circ. Physiol..

[32] Costelli P., De Tullio R., Baccino F.M., Melloni E. (2001). Activation of Ca 2+-dependent proteolysis in skeletal muscle and heart in cancer cachexia. Br. J. Cancer.

[33] Lee D.E., Brown J.L., Rosa-Caldwell M.E., Perry R.A., Brown L.A., Haynie W.S., Washington T.A., Wiggs M.P., Rajaram N., Greene N.P. (2021). Cancer-induced cardiac atrophy adversely affects myocardial redox state and mitochondrial oxidative characteristics. JCSM Rapid Commun..

[34] Busquets S., Fuster G., Ametller E., Olivan M., Figueras M., Costelli P., Carbó N., Argilés J.M., López-Soriano F.J. (2007). Resveratrol does not ameliorate muscle wasting in different types of cancer cachexia models. Clin. Nutr..

[35] Springer J., Tschirner A., Hartman K., Von Haehling S., Anker S.D., Doehner W. (2013). The xanthine oxidase inhibitor oxypurinol reduces cancer cachexia-induced cardiomyopathy. Int. J. Cardiol..

[36] Min K., Kwon O.S., Smuder A.J., Wiggs M.P., Sollanek K.J., Christou D.D., Yoo J.K., Hwang M.H., Szeto H.H., Kavazis A.N. (2015). Increased mitochondrial emission of reactive oxygen species and calpain activation are required for doxorubicin-induced cardiac and skeletal muscle myopathy. J. Physiol..

[37] Belloum Y., Rannou-Bekono F., Favier F.B. (2017). Cancer-induced cardiac cachexia: Pathogenesis and impact of physical activity. Oncol. Rep..

[38] Ino K., Shibata K., Kajiyama H., Yamamoto E., Nagasaka T., Nawa A., Nomura S., Kikkawa F. (2006). Angiotensin II type 1 receptor expression in ovarian cancer and its correlation with tumour angiogenesis and patient survival. Br. J. Cancer.

[39] Lever A.F., Hole D.J., Gillis C.R., McCallum I.R., McInnes G.T., MacKinnon P.L., Meredith P.A., Murray L.S., Reid J.L., Robertson J.W. (1998). Do inhibitors of angiotensin-I-converting enzyme protect against risk of cancer?. Lancet.

[40] Anker S.D., Negassa A., Coats A.J., Afzal R., Poole-Wilson P.A., Cohn J.N., Yusuf S. (2003). Prognostic importance of weight loss in chronic heart failure and the effect of treatment with angiotensin-converting-enzyme inhibitors: An observational study. Lancet.

[41] Delafontaine P., Akao M. (2006). Angiotensin II as candidate of cardiac cachexia. Curr. Opin. Clin. Nutr. Metab. Care.

[42] Shen C., Zhou J., Wang X., Yu X.-Y., Liang C., Liu B., Pan X., Zhao Q., Song J.L., Wang J. (2017). Angiotensin-II-induced muscle wasting is mediated by 25-hydroxycholesterol via GSK3β signaling pathway. EBioMedicine.

[43] Zablocki D., Sadoshima J. (2013). Angiotensin II and oxidative stress in the failing heart. Antioxid. Redox Signal..

[44] Zhang S., Lu Y., Jiang C. (2020). Inhibition of histone demethylase JMJD1C attenuates cardiac hypertrophy and fibrosis induced by angiotensin II. J. Recept. Signal Transduct..

[45] Jin D., Han F. (2020). FOXF1 ameliorates angiotensin II-induced cardiac fibrosis in cardiac fibroblasts through inhibiting the TGF-β1/Smad3 signaling pathway. J. Recept. Signal Transduct..

[46] Guan X.H., Hong X., Zhao N., Liu X.H., Xiao Y.F., Chen T.T., Deng L.B., Wang X.L., Wang J.B., Ji G.J. (2017). CD38 promotes angiotensin II-induced cardiac hypertrophy. J. Cell. Mol. Med..

[47] Yang L.-W., Qin D.-Z., James E., McKallip R.J., Wang N.-P., Zhang W.-W., Zheng R.-H., Han Q.-H., Zhao Z.-Q. (2019). CD44 deficiency in mice protects the heart against angiotensin II-induced cardiac fibrosis. Shock.

[48] Zheng C.-B., Gao W.-C., Xie M., Li Z., Ma X., Song W., Luo D., Huang Y., Yang J., Zhang P. (2021). Ang II promotes cardiac autophagy and hypertrophy via Orai1/STIM1. Front. Pharmacol..

[49] Cheng Z., Zhang M., Hu J., Lin J., Feng X., Wang S., Wang T., Gao E., Wang H., Sun D. (2018). Mst1 knockout enhances cardiomyocyte autophagic flux to alleviate angiotensin II-induced cardiac injury independent of angiotensin II receptors. J. Mol. Cell. Cardiol..

[50] Delafontaine P., Brink M. The growth hormone and insulin-like growth factor 1 axis in heart failure. Proceedings of the Annales D’endocrinologie.

[51] Rausch V., Sala V., Penna F., Porporato P.E., Ghigo A. (2021). Understanding the common mechanisms of heart and skeletal muscle wasting in cancer cachexia. Oncogenesis.

[52] Levine B.D. (2008). What do we know, and what do we still need to know?. J. Physiol..

[53] de Boer R.A., Hulot J.S., Tocchetti C.G., Aboumsallem J.P., Ameri P., Anker S.D., Bauersachs J., Bertero E., Coats A.J., Čelutkienė J. (2020). Common mechanistic pathways in cancer and heart failure. A scientific roadmap on behalf of the Translational Research Committee of the Heart Failure Association (HFA) of the European Society of Cardiology (ESC). Eur. J. Heart Fail..

[54] Scafoglieri A., Clarys J.P. (2018). Dual energy X-ray absorptiometry: Gold standard for muscle mass?. J. Cachexia Sarcopenia Muscle.

[55] Stimpson S.A., Leonard M.S., Clifton L.G., Poole J.C., Turner S.M., Shearer T.W., Remlinger K.S., Clark R.V., Hellerstein M.K., Evans W.J. (2013). Longitudinal changes in total body creatine pool size and skeletal muscle mass using the D3-creatine dilution method. J. Cachexia Sarcopenia Muscle.

[56] Bordignon C., Dos Santos B.S., Rosa D.D. (2022). Impact of Cancer Cachexia on Cardiac and Skeletal Muscle: Role of Exercise Training. Cancers.

[57] Skovgaard D., Hasbak P., Kjaer A. (2014). BNP predicts chemotherapy-related cardiotoxicity and death: Comparison with gated equilibrium radionuclide ventriculography. PLoS ONE.

[58] Penna F., Bonetto A., Muscaritoli M., Costamagna D., Minero V.G., Bonelli G., Fanelli F.R., Baccino F.M., Costelli P. (2010). Muscle atrophy in experimental cancer cachexia: Is the IGF-1 signaling pathway involved?. Int. J. Cancer.

[59] Silva F.B., Romero W.G., de Abreu Carvalho A.L.R., Souza G.A.A., Claudio E.R.G., Abreu G.R. (2017). Effects of treatment with chemotherapy and/or tamoxifen on the biomarkers of cardiac injury and oxidative stress in women with breast cancer. Medicine.

[60] Hulmi J.J., Nissinen T.A., Penna F., Bonetto A. (2021). Targeting the activin receptor signaling to counteract the multi-systemic complications of cancer and its treatments. Cells.

[61] Yndestad A., Ueland T., Øie E., Florholmen G., Halvorsen B., Attramadal H., Simonsen S., Frøland S.S., Gullestad L., Christensen G. (2004). Elevated levels of activin A in heart failure: Potential role in myocardial remodeling. Circulation.

[62] Pérez A.V., Doehner W., Von Haehling S., Schmidt H., Zimmermann A.V., Volk H.-D., Anker S.D., Rauchhaus M. (2010). The relationship between tumor necrosis factor-α, brain natriuretic peptide and atrial natriuretic peptide in patients with chronic heart failure. Int. J. Cardiol..

[63] Anker S.D., Chua T.P., Ponikowski P., Harrington D., Swan J.W., Kox W.J., Poole-Wilson P.A., Coats A.J. (1997). Hormonal changes and catabolic/anabolic imbalance in chronic heart failure and their importance for cardiac cachexia. Circulation.

[64] Martins T., Vitorino R., Amado F., Duarte J.A., Ferreira R. (2014). Biomarkers for cardiac cachexia: Reality or utopia. Clin. Chim. Acta.

[65] Schäfer M., Oeing C.U., Rohm M., Baysal-Temel E., Lehmann L.H., Bauer R., Volz H.C., Boutros M., Sohn D., Sticht C. (2016). Ataxin-10 is part of a cachexokine cocktail triggering cardiac metabolic dysfunction in cancer cachexia. Mol. Metab..

[66] Kazemi-Bajestani S.M., Becher H., Fassbender K., Chu Q., Baracos V.E. (2014). Concurrent evolution of cancer cachexia and heart failure: Bilateral effects exist. J. Cachexia Sarcopenia Muscle.

[67] Roderburg C., Loosen S.H., Jahn J.K., Gänsbacher J., Luedde T., Kostev K., Luedde M. (2021). Heart failure is associated with an increased incidence of cancer diagnoses. ESC Heart Fail..

[68] Burch G., Phillips J.H., Ansari A. (1968). The cachectic heart: A clinico-pathologic, electrocardiographic and roentgenographic entity. Dis. Chest.

[69] Springer J., Tschirner A., Haghikia A., Von Haehling S., Lal H., Grzesiak A., Kaschina E., Palus S., Pötsch M., Von Websky K. (2014). Prevention of liver cancer cachexia-induced cardiac wasting and heart failure. Eur. Heart J..

[70] Devine R.D., Bicer S., Reiser P.J., Velten M., Wold L.E. (2015). Metalloproteinase expression is altered in cardiac and skeletal muscle in cancer cachexia. Am. J. Physiol. Heart Circ. Physiol..

[71] Tian M., Nishijima Y., Asp M.L., Stout M.B., Reiser P.J., Belury M.A. (2010). Cardiac alterations in cancer-induced cachexia in mice. Int. J. Oncol..

[72] Trexler C.L., Odell A.T., Jeong M.Y., Dowell R.D., Leinwand L.A. (2017). Transcriptome and functional profile of cardiac myocytes is influenced by biological sex. Circ. Cardiovasc. Genet..

[73] Pin F., Barreto R., Kitase Y., Mitra S., Erne C.E., Novinger L.J., Zimmers T.A., Couch M.E., Bonewald L.F., Bonetto A. (2018). Growth of ovarian cancer xenografts causes loss of muscle and bone mass: A new model for the study of cancer cachexia. J. Cachexia Sarcopenia Muscle.

[74] Berent T.E., Dorschner J.M., Meyer T., Craig T.A., Wang X., Kunz H., Jatoi A., Lanza I.R., Chen H., Kumar R. (2019). Impaired cardiac performance, protein synthesis, and mitochondrial function in tumor-bearing mice. PLoS ONE.

[75] Cramer L., Hildebrandt B., Kung T., Wichmann K., Springer J., Doehner W., Sandek A., Valentova M., Stojakovic T., Scharnagl H. (2014). Cardiovascular function and predictors of exercise capacity in patients with colorectal cancer. J. Am. Coll. Cardiol..

[76] Danese E., Montagnana M., Giudici S., Aloe R., Franchi M., Guidi G.C., Lippi G. (2013). Highly-sensitive troponin I is increased in patients with gynecological cancers. Clin. Biochem..

[77] Pavo N., Raderer M., Hülsmann M., Neuhold S., Adlbrecht C., Strunk G., Goliasch G., Gisslinger H., Steger G.G., Hejna M. (2015). Cardiovascular biomarkers in patients with cancer and their association with all-cause mortality. Heart.

[78] Labib D., Satriano A., Dykstra S., Hansen R., Mikami Y., Guzzardi D.G., Slavikova Z., Feuchter P., Flewitt J., Rivest S. (2021). Effect of Active Cancer on the Cardiac Phenotype: A Cardiac Magnetic Resonance Imaging-Based Study of Myocardial Tissue Health and Deformation in Patients With Chemotherapy-Naïve Cancer. J. Am. Heart Assoc..

[79] Stoltzfus K.C., Zhang Y., Sturgeon K., Sinoway L.I., Trifiletti D.M., Chinchilli V.M., Zaorsky N.G. (2020). Fatal heart disease among cancer patients. Nat. Commun..

[80] Tadic M., Genger M., Baudisch A., Kelle S., Cuspidi C., Belyavskiy E., Burkhardt F., Venneri L., Attanasio P., Pieske B. (2018). Left ventricular strain in chemotherapy-naive and radiotherapy-naive patients with cancer. Can. J. Cardiol..

[81] Tadic M., Baudisch A., Haßfeld S., Heinzel F., Cuspidi C., Burkhardt F., Escher F., Attanasio P., Pieske B., Genger M. (2018). Right ventricular function and mechanics in chemotherapy-and radiotherapy-naïve cancer patients. Int. J. Cardiovasc. Imaging.

[82] Tadic M., Genger M., Cuspidi C., Belyavskiy E., Frydas A., Dordevic A., Morris D.A., Völkl J., Parwani A.S., Pieske B. (2019). Phasic left atrial function in cancer patients before initiation of anti-cancer therapy. J. Clin. Med..

[83] Von Hoff D.D., Layard M.W., Basa P., Davis H.L., Von Hoff A.L., Rozencweig M., Muggia F.M. (1979). Risk factors for doxorubicin-lnduced congestive heart failure. Ann. Intern. Med..

[84] de Boer R.A., Aboumsallem J.P., Bracun V., Leedy D., Cheng R., Patel S., Rayan D., Zaharova S., Rymer J., Kwan J.M. (2021). A new classification of cardio-oncology syndromes. Cardio-Oncology.

[85] Huot J.R., Essex A.L., Gutierrez M., Barreto R., Wang M., Waning D.L., Plotkin L.I., Bonetto A. (2019). Chronic treatment with multi-kinase inhibitors causes differential toxicities on skeletal and cardiac muscles. Cancers.

[86] Pouna P., Bonoron-Adèle S., Gouverneur G., Tariosse L., Besse P., Robert J. (1996). Development of the model of rat isolated perfused heart for the evaluation of anthracycline cardiotoxicity and its circumvention. Br. J. Pharmacol..

[87] Platel D., Pouna P., Bonoron-Adèle S., Robert J. (2000). Preclinical evaluation of the cardiotoxicity of taxane–anthracycline combinations using the model of isolated perfused rat heart. Toxicol. Appl. Pharmacol..

[88] Hasinoff B.B. (2010). The cardiotoxicity and myocyte damage caused by small molecule anticancer tyrosine kinase inhibitors is correlated with lack of target specificity. Toxicol. Appl. Pharmacol..

[89] Albini A., Festa M.M., Ring N., Baci D., Rehman M., Finzi G., Sessa F., Zacchigna S., Bruno A., Noonan D.M. (2021). A polyphenol-rich extract of Olive Mill Wastewater Enhances cancer chemotherapy effects, while mitigating cardiac toxicity. Front. Pharmacol..

[90] Knapik-Czajka M., Jurczyk M., Bieleń J., Aleksandrovych V., Gawędzka A., Stach P., Drąg J., Gil K. (2021). Effect of 5-fluorouracil on branched-chain α-keto acid dehydrogenase (BCKDH) complex in rat’s heart. Folia Med. Crac..

[91] Li Y., Zhang Y., Zhou X., Lei X., Li X., Wei L. (2021). Dynamic observation of 5-fluorouracil-induced myocardial injury and mitochondrial autophagy in aging rats. Exp. Ther. Med..

[92] Shi H., Tang H., Ai W., Zeng Q., Yang H., Zhu F., Wei Y., Feng R., Wen L., Pu P. (2021). Schisandrin B Antagonizes Cardiotoxicity Induced by Pirarubicin by Inhibiting Mitochondrial Permeability Transition Pore (mPTP) Opening and Decreasing Cardiomyocyte Apoptosis. Front. Pharmacol..

[93] El-Hawwary A.A., Omar N.M. (2019). The influence of ginger administration on cisplatin-induced cardiotoxicity in rat: Light and electron microscopic study. Acta Histochem..

[94] Saleh D.O., Mansour D.F., Mostafa R.E. (2020). Rosuvastatin and simvastatin attenuate cisplatin-induced cardiotoxicity via disruption of endoplasmic reticulum stress-mediated apoptotic death in rats: Targeting ER-chaperone GRP78 and calpain-1 pathways. Toxicol. Rep..

[95] Abd-ElRaouf A., Nada A.S., Mohammed N.E.-D.A., Amer H.A., Abd-ElRahman S.S., Abdelsalam R.M., Salem H.A. (2021). Low dose gamma irradiation attenuates cyclophosphamide-induced cardiotoxicity in rats: Role of NF-κB signaling pathway. Int. J. Radiat. Biol..

[96] Elrashidy R.A., Hasan R.A. (2021). Cilostazol preconditioning alleviates cyclophosphamide-induced cardiotoxicity in male rats: Mechanistic insights into SIRT1 signaling pathway. Life Sci..

[97] Kamel S.S., Baky N.A.A., Karkeet R.M., Osman A.M.M., Sayed-Ahmed M.M., Fouad M.A. (2022). Astaxanthin extenuates the inhibition of aldehyde dehydrogenase and Klotho protein expression in cyclophosphamide-induced acute cardiomyopathic rat model. Clin. Exp. Pharmacol. Physiol..

[98] Al-Taher A.Y., Morsy M.A., Rifaai R.A., Zenhom N.M., Abdel-Gaber S.A. (2020). Paeonol attenuates methotrexate-induced cardiac toxicity in rats by inhibiting oxidative stress and suppressing TLR4-induced NF-κB inflammatory pathway. Mediat. Inflamm..

[99] Mohan N., Shen Y., Endo Y., ElZarrad M.K., Wu W.J. (2016). Trastuzumab, but not pertuzumab, dysregulates HER2 signaling to mediate inhibition of autophagy and increase in reactive oxygen species production in human cardiomyocytes. Mol. Cancer Ther..

[100] Novo G., Di Lisi D., Bronte E., Macaione F., Accurso V., Badalamenti G., Rinaldi G., Siragusa S., Novo S., Russo A. (2020). Cardiovascular toxicity in cancer patients treated with tyrosine kinase inhibitors: A real-world single-center experience. Oncology.

[101] Hunt S.A., Abraham W.T., Chin M.H., Feldman A.M., Francis G.S., Ganiats T.G., Jessup M., Konstam M.A., Mancini D.M., Michl K. (2009). 2009 focused update incorporated into the ACC/AHA 2005 guidelines for the diagnosis and management of heart failure in adults: A report of the American College of Cardiology Foundation/American Heart Association Task Force on Practice Guidelines developed in collaboration with the International Society for Heart and Lung Transplantation. J. Am. Coll. Cardiol..

[102] Boyd A., Stoodley P., Richards D., Hui R., Harnett P., Vo K., Marwick T., Thomas L. (2017). Anthracyclines induce early changes in left ventricular systolic and diastolic function: A single centre study. PLoS ONE.

[103] Serrano J.M., González I., Del Castillo S., Muñiz J., Morales L.J., Moreno F., Jiménez R., Cristóbal C., Graupner C., Talavera P. (2015). Diastolic dysfunction following anthracycline-based chemotherapy in breast cancer patients: Incidence and predictors. Oncologist.

[104] Ito M., Horimoto Y., Sasaki R., Miyazaki S., Orihata G., Saito M. (2021). Cardiotoxicity after Additional Administration of Pertuzumab following Long-Term Trastuzumab: Report of 2 Cases. Case Rep. Oncol..

[105] Gianni L., Lladó A., Bianchi G., Cortes J., Kellokumpu-Lehtinen P.-L., Cameron D.A., Miles D., Salvagni S., Wardley A., Goeminne J.-C. (2010). Open-label, phase II, multicenter, randomized study of the efficacy and safety of two dose levels of pertuzumab, a human epidermal growth factor receptor 2 dimerization inhibitor, in patients with human epidermal growth factor receptor 2–negative metastatic breast cancer. J. Clin. Oncol..

[106] Mahmood S.S., Fradley M.G., Cohen J.V., Nohria A., Reynolds K.L., Heinzerling L.M., Sullivan R.J., Damrongwatanasuk R., Chen C.L., Gupta D. (2018). Myocarditis in patients treated with immune checkpoint inhibitors. J. Am. Coll. Cardiol..

[107] Witte K.K., Clark A.L., Cleland J.G. (2001). Chronic heart failure and micronutrients. J. Am. Coll. Cardiol..

[108] Engelen M., Safar A., Bartter T., Koeman F., Deutz N. (2015). High anabolic potential of essential amino acid mixtures in advanced nonsmall cell lung cancer. Ann. Oncol..

[109] Nystoriak M.A., Bhatnagar A. (2018). Cardiovascular effects and benefits of exercise. Front. Cardiovasc. Med..

[110] Parry T.L., Hayward R. (2018). Exercise Protects against Cancer-induced Cardiac Cachexia. Med. Sci. Sports Exerc..

[111] Fernandes L., Tobias G., Paixão A., Dourado P., Voltarelli V., Brum P. (2020). Exercise training delays cardiac remodeling in a mouse model of cancer cachexia. Life Sci..

[112] Padrão A.I., Nogueira-Ferreira R., Vitorino R., Carvalho D., Correia C., Neuparth M.J., Pires M.J., Faustino-Rocha A.I., Santos L.L., Oliveira P.A. (2018). Exercise training protects against cancer-induced cardiac remodeling in an animal model of urothelial carcinoma. Arch. Biochem. Biophys..

[113] MacVicar M.G., Winningham M.L., Nickel J.L. (1989). Effects of aerobic interval training on cancer patients’ functional capacity. Nurs. Res..

[114] Courneya K.S., Segal R.J., Mackey J.R., Gelmon K., Reid R.D., Friedenreich C.M., Ladha A.B., Proulx C., Vallance J., Lane K. (2007). Effects of aerobic and resistance exercise in breast cancer patients receiving adjuvant chemotherapy: A multicenter randomized controlled trial. J. Clin. Oncol..

[115] Courneya K.S., McKenzie D.C., Mackey J.R., Gelmon K., Friedenreich C.M., Yasui Y., Reid R.D., Cook D., Jespersen D., Proulx C. (2013). Effects of exercise dose and type during breast cancer chemotherapy: Multicenter randomized trial. J. Natl. Cancer Inst..

[116] Van Waart H., Stuiver M.M., van Harten W.H., Geleijn E., Kieffer J.M., Buffart L.M., de Maaker-Berkhof M., Boven E., Schrama J., Geenen M.M. (2015). Effect of low-intensity physical activity and moderate-to high-intensity physical exercise during adjuvant chemotherapy on physical fitness, fatigue, and chemotherapy completion rates: Results of the PACES randomized clinical trial. J. Clin. Oncol..

[117] Scott J.M., Iyengar N.M., Nilsen T.S., Michalski M., Thomas S.M., Herndon J., Sasso J., Yu A., Chandarlapaty S., Dang C.T. (2018). Feasibility, safety, and efficacy of aerobic training in pretreated patients with metastatic breast cancer: A randomized controlled trial. Cancer.

[118] Courneya K.S., Sellar C.M., Stevinson C., McNeely M.L., Peddle C.J., Friedenreich C.M., Tankel K., Basi S., Chua N., Mazurek A. (2009). Randomized controlled trial of the effects of aerobic exercise on physical functioning and quality of life in lymphoma patients. J. Clin. Oncol..

[119] Segal R.J., Reid R.D., Courneya K.S., Sigal R.J., Kenny G.P., Prud’Homme D.G., Malone S.C., Wells G.A., Scott C.G., Slovinec D’Angelo M.E. (2009). Randomized controlled trial of resistance or aerobic exercise in men receiving radiation therapy for prostate cancer. J. Clin. Oncol..

[120] Pinto B., Stein K., Dunsiger S. (2013). Peer Mentoring to Promote Exercise Among Cancer Survivors: A Community Partnership: S12-1. Pscyho-Oncology.

[121] Rogers L.Q., Courneya K.S., Anton P.M., Hopkins-Price P., Verhulst S., Vicari S.K., Robbs R.S., Mocharnuk R., McAuley E. (2015). Effects of the BEAT Cancer physical activity behavior change intervention on physical activity, aerobic fitness, and quality of life in breast cancer survivors: A multicenter randomized controlled trial. Breast Cancer Res. Treat..

[122] Jones L.W., Douglas P.S., Khouri M.G., Mackey J.R., Wojdyla D., Kraus W.E., Whellan D.J., O’Connor C.M. (2014). Safety and efficacy of aerobic training in patients with cancer who have heart failure: An analysis of the HF-ACTION randomized trial. J. Clin. Oncol..

[123] Hong D.S., Hui D., Bruera E., Janku F., Naing A., Falchook G.S., Piha-Paul S., Wheler J.J., Fu S., Tsimberidou A.M. (2014). MABp1, a first-in-class true human antibody targeting interleukin-1α in refractory cancers: An open-label, phase 1 dose-escalation and expansion study. Lancet Oncol..

[124] Lust J.A., Lacy M.Q., Zeldenrust S.R., Dispenzieri A., Gertz M.A., Witzig T.E., Kumar S., Hayman S.R., Russell S.J., Buadi F.K. (2009). Induction of a chronic disease state in patients with smoldering or indolent multiple myeloma by targeting interleukin 1β-induced interleukin 6 production and the myeloma proliferative component. Mayo Clin. Proc..

[125] Jatoi A., Ritter H.L., Dueck A., Nguyen P.L., Nikcevich D.A., Luyun R.F., Mattar B.I., Loprinzi C.L. (2010). A placebo-controlled, double-blind trial of infliximab for cancer-associated weight loss in elderly and/or poor performance non-small cell lung cancer patients (N01C9). Lung Cancer.

[126] Bayliss T.J., Smith J.T., Schuster M., Dragnev K.H., Rigas J.R. (2011). A humanized anti-IL-6 antibody (ALD518) in non-small cell lung cancer. Expert Opin. Biol. Ther..

[127] Kwon H.J., Cot T.R., Cuffe M.S., Kramer J.M., Braun M.M. (2003). Case reports of heart failure after therapy with a tumor necrosis factor antagonist. Ann. Intern. Med..

[128] Mantovani G., Macciò A., Madeddu C., Serpe R., Antoni G., Massa E., Dessì M., Panzone F. (2010). Phase II nonrandomized study of the efficacy and safety of COX-2 inhibitor celecoxib on patients with cancer cachexia. J. Mol. Med..

[129] Reid J., Hughes C., Murray L., Parsons C., Cantwell M. (2013). Non-steroidal anti-inflammatory drugs for the treatment of cancer cachexia: A systematic review. Palliat. Med..

[130] Solheim T.S., Fearon K.C., Blum D., Kaasa S. (2013). Non-steroidal anti-inflammatory treatment in cancer cachexia: A systematic literature review. Acta Oncol..

[131] Musolino V., Palus S., Tschirner A., Drescher C., Gliozzi M., Carresi C., Vitale C., Muscoli C., Doehner W., von Haehling S. (2016). Megestrol acetate improves cardiac function in a model of cancer cachexia-induced cardiomyopathy by autophagic modulation. J. Cachexia Sarcopenia Muscle.

[132] Garcia V.R., López-Briz E., Sanchis R.C., Perales J.L.G., Bort-Martí S. (2013). Megestrol acetate for treatment of anorexia-cachexia syndrome. Cochrane Database Syst. Rev..

[133] Loprinzi C.L., Kugler J.W., Sloan J.A., Mailliard J.A., Krook J.E., Wilwerding M.B., Rowland K.M., Camoriano J.K., Novotny P.J., Christensen B.J. (1999). Randomized comparison of megestrol acetate versus dexamethasone versus fluoxymesterone for the treatment of cancer anorexia/cachexia. J. Clin. Oncol..

[134] Moertel C., Schutt A., Reitemeier R., Hahn R. (1974). Corticosteroid therapy of preterminal gastrointestinal cancer. Cancer.

[135] Yavuzsen T., Davis M.P., Walsh D., LeGrand S., Lagman R. (2005). Systematic review of the treatment of cancer-associated anorexia and weight loss. Database Abstr. Rev. Eff. Qual. Assess. Rev..

[136] Temel J.S., Abernethy A.P., Currow D.C., Friend J., Duus E.M., Yan Y., Fearon K.C. (2016). Anamorelin in patients with non-small-cell lung cancer and cachexia (ROMANA 1 and ROMANA 2): Results from two randomised, double-blind, phase 3 trials. Lancet Oncol..

[137] Roeland E.J., Bohlke K., Baracos V.E., Bruera E., Del Fabbro E., Dixon S., Fallon M., Herrstedt J., Lau H., Platek M. (2020). Management of cancer cachexia: ASCO guideline. J. Clin. Oncol..

[138] Reiche E.M.V., Nunes S.O.V., Morimoto H.K. (2004). Stress, depression, the immune system, and cancer. Lancet Oncol..

[139] Elkina Y., Palus S., Tschirner A., Hartmann K., Von Haehling S., Doehner W., Mayer U., Coats A.J., Beadle J., Anker S.D. (2013). Tandospirone reduces wasting and improves cardiac function in experimental cancer cachexia. Int. J. Cardiol..

[140] Devine R.D., Eichenseer C.M., Wold L.E. (2016). Minocycline attenuates cardiac dysfunction in tumor-burdened mice. J. Mol. Cell. Cardiol..

[141] Palus S., Von Haehling S., Flach V.C., Tschirner A., Doehner W., Anker S.D., Springer J. (2013). Simvastatin reduces wasting and improves cardiac function as well as outcome in experimental cancer cachexia. Int. J. Cardiol..

[142] Muscaritoli M., Costelli P., Bossola M., Grieco G., Bonelli G., Bellantone R., Doglietto G.B., Rossi-Fanelli F., Baccino F.M. (2003). Effects of simvastatin administration in an experimental model of cancer cachexia. Nutrition.

[143] Saitoh M., Hatanaka M., Konishi M., Ishida J., Palus S., Ebner N., Döhner W., von Haehling S., Anker S.D., Springer J. (2016). Erythropoietin improves cardiac wasting and outcomes in a rat model of liver cancer cachexia. Int. J. Cardiol..

[144] Aguilar D., Strom J., Chen Q.M. (2014). Glucocorticoid induced leucine zipper inhibits apoptosis of cardiomyocytes by doxorubicin. Toxicol. Appl. Pharmacol..

[145] Legi A., Rodriguez E., Eckols T.K., Mistry C., Robinson P. (2021). Substance P antagonism prevents chemotherapy-induced cardiotoxicity. Cancers.

[146] Find a Study. https://clinicaltrials.gov/ct2/home.

[147] Cardinale D., Sandri M.T., Martinoni A., Tricca LabTech A., Civelli M., Lamantia G., Cinieri S., Martinelli G., Cipolla C.M., Fiorentini C. (2000). Left ventricular dysfunction predicted by early troponin I release after high-dose chemotherapy. J. Am. Coll. Cardiol..

[148] Avila M.S., Ayub-Ferreira S.M., de Barros Wanderley M.R., das Dores Cruz F., Gonçalves Brandão S.M., Rigaud V.O.C., Higuchi-dos-Santos M.H., Hajjar L.A., Kalil Filho R., Hoff P.M. (2018). Carvedilol for prevention of chemotherapy-related cardiotoxicity: The CECCY trial. J. Am. Coll. Cardiol..

[149] Straughn A.R., Kelm N.Q., Kakar S.S. (2021). Withaferin A and Ovarian Cancer Antagonistically Regulate Skeletal Muscle Mass. Front. Cell Dev. Biol..

[150] Calin G.A., Dumitru C.D., Shimizu M., Bichi R., Zupo S., Noch E., Aldler H., Rattan S., Keating M., Rai K. (2002). Frequent deletions and down-regulation of micro-RNA genes miR15 and miR16 at 13q14 in chronic lymphocytic leukemia. Proc. Natl. Acad. Sci. USA.

[151] He W.A., Calore F., Londhe P., Canella A., Guttridge D.C., Croce C.M. (2014). Microvesicles containing miRNAs promote muscle cell death in cancer cachexia via TLR7. Proc. Natl. Acad. Sci. USA.

[152] Thum T., Gross C., Fiedler J., Fischer T., Kissler S., Bussen M., Galuppo P., Just S., Rottbauer W., Frantz S. (2008). MicroRNA-21 contributes to myocardial disease by stimulating MAP kinase signalling in fibroblasts. Nature.

[153] Bei Y., Xiao J. (2017). MicroRNAs in muscle wasting and cachexia induced by heart failure. Nat. Rev. Cardiol..

[154] Bindels L.B., Neyrinck A.M., Loumaye A., Catry E., Walgrave H., Cherbuy C., Leclercq S., Van Hul M., Plovier H., Pachikian B. (2018). Increased gut permeability in cancer cachexia: Mechanisms and clinical relevance. Oncotarget.

[155] Jiang Y., Guo C., Zhang D., Zhang J., Wang X., Geng C. (2014). The altered tight junctions: An important gateway of bacterial translocation in cachexia patients with advanced gastric cancer. J. Interferon Cytokine Res..

[156] Bindels L.B., Beck R., Schakman O., Martin J.C., De Backer F., Sohet F.M., Dewulf E.M., Pachikian B.D., Neyrinck A.M., Thissen J.-P. (2012). Restoring specific lactobacilli levels decreases inflammation and muscle atrophy markers in an acute leukemia mouse model. PLoS ONE.

[157] Tang W.W., Li D.Y., Hazen S.L. (2019). Dietary metabolism, the gut microbiome, and heart failure. Nat. Rev. Cardiol..

